# Using a Mobile App–Based Video Recommender System of Patient Narratives to Prepare Women for Breast Cancer Surgery: Development and Usability Study Informed by Qualitative Data

**DOI:** 10.2196/22970

**Published:** 2021-06-02

**Authors:** Ilja Ormel, Charles C Onu, Mona Magalhaes, Terence Tang, John B Hughes, Susan Law

**Affiliations:** 1 St Mary's Research Centre Montreal, QC Canada; 2 Department of Family Medicine McGill University Montréal, QC Canada; 3 School of Computer Science McGill University Montréal, QC Canada; 4 Trillium Health Partners Mississauga, ON Canada; 5 McGill University Montréal, QC Canada; 6 Institute for Health Policy, Management and Evaluation University of Toronto Toronto, ON Canada

**Keywords:** qualitative research, illness narratives, experiential information, breast cancer, surgery, tailored information, recommender system, patient information and communication, mobile app, mobile phone

## Abstract

**Background:**

Women diagnosed with breast cancer are often bombarded with information. Such information overload can lead to misunderstandings and hamper women’s capacity for making informed decisions about their care. For women with breast cancer, this uncertainty is particularly severe in the period before surgery. Personalized narratives about others’ experiences can help patients better understand the disease course, the quality and type of care to be expected, the clinical decision-making processes, and the strategies for coping. Existing resources and eHealth apps rarely include experiential information, and no tools exist that tailor information for individual preferences and needs—offering the right information at the right time and in the right format. Combining high-quality experiential evidence with novel technical approaches may contribute to patient-centered solutions in this area.

**Objective:**

This study aims to design and seek preliminary feedback on a mobile app that will improve information access about surgery for patients with breast cancer, by drawing on a qualitative collection of personal narratives from a diverse sample of Canadian women and using video and audio recordings or audio recordings from the Canadian Health Experiences Research Network.

**Methods:**

In a previous study, we conducted in-depth interviews with 35 Canadian women and used video and audio recordings or audio recordings to collect stories about the lived experiences of breast cancer. The participants highlighted the need for more specific information between diagnosis and surgery that was relevant to their personal situations and preferences. They also wanted to learn from other women’s experiences. We worked with patients, clinicians, and informatics experts to develop a mobile app that provides access to tailored experiential information relevant to women’s personal situations and preferences. We completed focus groups and qualitative interviews, conducted a further analysis of the original qualitative data, designed novel software using artificial intelligence, and sought preliminary feedback from users on a new app via focus groups and a survey.

**Results:**

The secondary analysis of the breast cancer narratives revealed key themes and their interconnections relevant to the experience of surgery, including preparation, treatment decisions, aftercare, reconstruction, prostheses, lumpectomy and mastectomy, and complications. These themes informed the development of the structure and content of the app. We developed a recommender system within the app by using content matching (user and speaker profiles and user interests and video content) and collaborative filtering to identify clips marked as relevant by the user and by similar users. A 2-minute animated introductory video for users was developed. Pilot testing revealed generally positive responses regarding the content and value of this type of e-tool.

**Conclusions:**

Developing reliable, evidence-based tools and apps that are based on diverse collections of people’s experiences of illness offers a novel approach to help manage the plethora of information that women face after a diagnosis of breast cancer.

## Introduction

### Background

The last few decades have been marked by the rapid expansion of web-based health information [1], and breast cancer has been noted as the most searched for health topic on the web [2]. This has not only helped to promote the availability of relevant breast cancer information but also exacerbated challenges related to information overload [3]. Therefore, women with breast cancer are particularly vulnerable to experience information overload, which is associated with a negative impact on patients’ treatment or behavioral decisions [3-6] and associated with anxiety and distress [6]. In biomedical research, the information bottleneck has shifted from data collection to data management and analysis [7], and it appears that there is a parallel shift regarding information for patients. Tailoring information to individual needs can better support patients in their search and retrieval efforts for securing *appropriate* information [8]. This will not only reduce information overload but also ensure that patients do not miss important treatment information, which is another factor that negatively impacts the health and well-being of women with breast cancer [9,10].

Mobile apps offer innovative solutions for improving health care for various health conditions [11]. Several of these apps include the provision of tailored information [12-15]. This innovation has evolved rapidly in the last decade, and it is estimated that by 2018, nearly 2 billion smartphone and tablet users accessed health care–related apps [16].

### Experiential Information in Apps

A recent systematic review of empirical studies on mobile apps (n=29) for breast cancer care [17] and another review on the evidence for mobile app use (n=9) during the treatment of breast cancer [11] identified only one study that included access to experiential information in the form of a personal story forum containing five recorded stories [18]. This is despite evidence that patients turn to the internet and other sources to purposefully search for experiential information [19]. However, the quality and access to this type of information is highly variable, and there is a need to provide rigorously developed, reliable, and tailored information regarding the experiences of others with similar conditions. Experiences of other patients are an important part of the evidence base that is available to patients; experiential evidence increases awareness of various treatment options, normalizes aspects of illness and treatment, and supports and informs decision making. Dismissing personal stories as *anecdotes* is a serious misunderstanding [20]. To date, research findings underscore the value and impact of patients’ exposure to experiential information, which includes providing comfort and ensuring a more realistic outlook about the future [21]. Similarly, Ziebland and Wyke [19] reported that experiential information can support people in making better health care choices, raising awareness of certain health issues, improving health literacy, comparing each other’s situation, and accessing more appropriate services. From our study on women’s experiences of breast cancer, we found that experiential information can complement women’s information needs about subjects that are not always communicated through factual and biomedical types of information [22]. It is argued that the inclusion of experiential health information will remain a key feature of eHealth strategies because of the appeal and memorability of stories and the need to make contact with peers [19]. However, there are concerns regarding people’s reliance on the internet for experiential information and the quality and reliability of this information [19]. A review of existing apps demonstrated that such resources are scarce. Few of them are evidence-based and many are misleading [23], as apps are often introduced into clinical care before benefits and risks for patients and health care professionals are evaluated [11]. Both the systematic reviews mentioned earlier found that rigorous trials in this area are lacking.

Women not only need to receive the right information at the right time and in the right format but also need to receive information from trustworthy sources, such as health care professionals, patient support organizations, and other patients, especially when information is offered on the web or through a mobile app. The challenge lies in developing evidence-based information tools that respond to women’s personal information needs. Such tools are relatively rare to date, and to our knowledge, no tools exist that provide information that is *tailored* to individual preferences and needs**.** Greater insights regarding women’s needs at particular times in their cancer journeys and regarding the technical requirements for such a tool would contribute to novel solutions. To our knowledge, there are no tools yet that help patients understand how to prepare for breast cancer surgery based on lived experiences and, in particular, that draw from experiential evidence gathered through rigorous qualitative methods. This paper reports on the development of a new tailored information app called Health Experiences and Real Stories (HERS) that allows women with newly diagnosed breast cancer to retrieve information (in English or French) from an existing database of women’s experiences with breast cancer. One of the key findings from previous studies was that women missed important information during the diagnostic phase, especially while preparing for surgery. However, women also reported that they struggled to handle information at the start of their breast cancer journey [22]. Women with suspicious lumps or other symptoms of potential breast cancer may often be seen and treated by a surgeon before they come under the care of a specialized breast cancer team (providing multidisciplinary care and support throughout the *journey* [8]). This diagnostic stage is typically described as one of the most bewildering periods [8,24] when women are usually coming to terms with this new disease and may be seeking information about breast cancer, treatments, and options [25].

### Objective

This study aims to contribute to the development of tools that provide patients with tailored experiential information based on rigorous qualitative research. In the following sections, we describe the work undertaken in four phases to conceptualize, develop, and obtain preliminary feedback from users for the HERS app.

## Methods

### Overview

**Figure 1:**
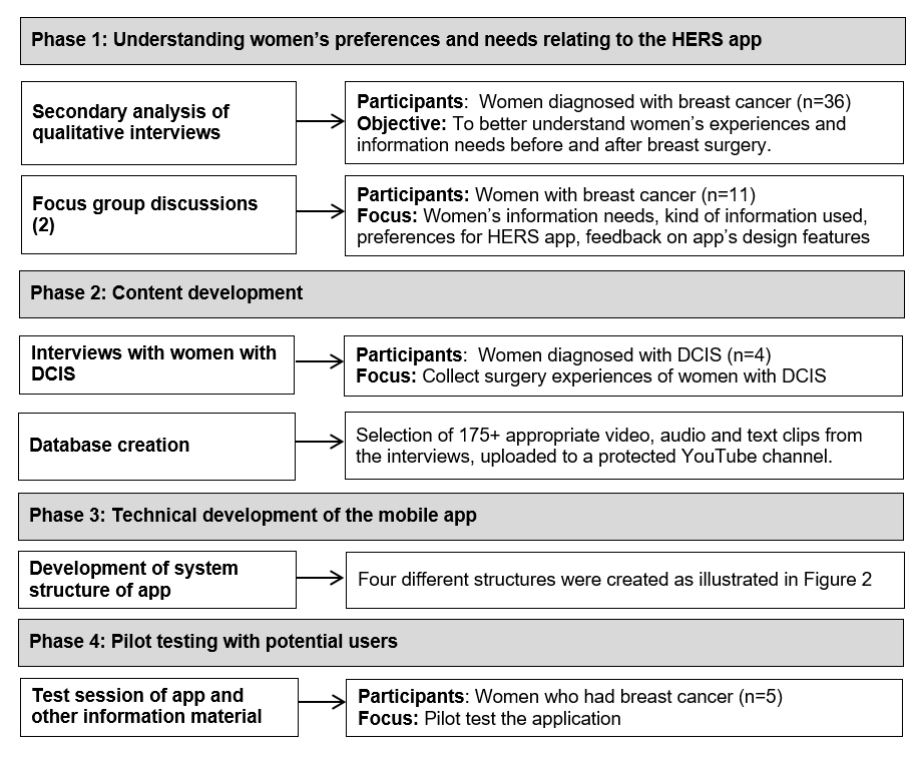
Process of the Health Experiences and Real Stories app development. DCIS: ductal carcinoma in situ; HERS: Health Experiences and Real Stories.

Our multidisciplinary research team (clinicians, computer engineers, and researchers) worked in close collaboration with an expert advisory panel (patients, representatives of breast cancer organizations, and clinicians) to develop and test the HERS app over the study period from July 2016 to June 2018. The four phases of work consisted of understanding women’s preferences and needs related to the app (phase 1), content development (phase 2), technical development (phase 3), and pilot testing (phase 4), as presented in Figure 1. The research ethics committee of Saint Mary’s Hospital Center (SMHC) in Montreal, Quebec, Canada, approved the study in June 2016 (reference number 11-22 B, amendment 3). Participants for the interviews and focus groups were recruited through breast cancer patient and community organizations and support groups, personal networks, our expert advisory committee, and social media. Informed consent was obtained from all participants.

### Phase 1: Understanding Women’s Preferences and Needs Relating to the HERS App

A secondary analysis of the transcripts from 35 interviews previously conducted with women with breast cancer was undertaken to identify women’s information needs before and after surgery. This analysis informed the questions developed for the focus group discussions in this study. Details regarding the methods for the original qualitative research have been published elsewhere [22].

Two focus groups involving women with experiences of breast cancer were convened in July 2016 (n=6) and September 2016 (n=5) to (1) consider information needs (helpful information, information needs, and lack of information) related to surgery, (2) review sample videos to understand the value of experiential information, and (3) better understand their preferences regarding design features of a tailored information app. In focus group 2 (different participants), we presented a summary of the results of focus group 1 and discussed the findings further. The women were aged between 43 and 65 years. The participants had been diagnosed with breast cancer and had undergone various surgeries (lumpectomy, single or double mastectomy, and reconstruction). Both focus groups were facilitated by the principal investigator, senior qualitative researcher, and computer scientists.

### Phase 2: Content Development

The content for this app was drawn primarily from the data collected in previous qualitative research completed by our team regarding the experiences of 35 Canadian women with breast cancer (Canadian Women’s Experiences with Breast Cancer study). These results are presented on the web (Canadian Health Experiences Research Network, 2021 [26]) where we created topic summaries, including illustrative video and audio clips, from our analysis of issues important to the women that we interviewed. Given that we had previously excluded women diagnosed with the earliest form of breast cancer—ductal carcinoma in situ (DCIS), [22]—because of the particular definition of breast cancer adopted for the original collection and the fact that this group also underwent breast surgery (patients with DCIS would therefore be inclusive of potential users of the app), we conducted four additional interviews with women diagnosed with DCIS between October 2016 and September 2017 using the same methods for collecting narrative interviews as per the original study. Women were asked to share their experiences with breast cancer beginning with an open narrative question (“Can you tell me about your experiences with breast cancer from the beginning up until now?”) followed by a semistructured set of questions. Participants in the narrative interviews had already consented to the future use of their interview materials (transcript, audio, and video recordings), which included apps such as web-based resources and for research and teaching; all participants were provided with a transcript of their interview for review.

Qualitative analysis of the four DCIS interviews was conducted, along with a secondary analysis of the original 35 interviews (36 interviews were conducted but one participant withdrew from the study) of women with breast cancer, focusing on women’s experiences of breast surgery. Using the framework method [27], we selected text from the 39 interviews that were specifically related to breast surgery, such as experiences with surgical procedures, but also topics such as body image, talking to children, sexuality, and information needs. Two senior qualitative researchers analyzed the data, using the analytic software NVivo 10 (QSR International), and then developed a list of relevant topics relating to content (in collaboration with the principal investigator and computer engineers) for the HERS app based on the themes and categories that emerged from the coding framework.

### Phase 3: Technical Development of the Mobile App

Working closely with an expert advisory panel and women diagnosed with breast cancer, we built the HERS app based on the needs and preferences of women with breast cancer for tailored information from phase 1 with content from phase 2. We developed the HERS app as a mobile app for Android smartphones or tablets in the Java programming language. The content (video clips) is housed on a YouTube platform on the web. To provide tailored information to users, we developed a recommendation engine as a web service (based on the representational state transfer protocol) powered by a Microsoft structured query language database that stores user information and metadata of available videos. The mobile app accesses the web service and retrieves appropriate content from YouTube to display to users (Multimedia Appendix 1).

After completion of the prototype app, we made further improvements and iterations based on the feedback from a test session (testing the app, focus group, and surveys), as described in phase 4.

### Phase 4: Obtaining Preliminary Feedback From Potential Users

The test session took place in June 2018 with 5 women (aged 51-66 years with a minimum of high school education), who had been diagnosed with breast cancer between 2005 and 2016. Two of the women had a recurrence of cancer since their diagnosis and 2 women previously participated in the focus group of phase 1. The goals of the session were as follows:

- To consider women’s responses to existing web-based resources for breast surgery that contain more factual information and to the pilot version of the HERS app presenting experiential information
- To gather perspectives on the HERS mobile app regarding what was useful and to gather any recommendations for improvement

The women first explored the information resources offered by the app. These included resources offered by reliable breast cancer organizations such as the Canadian Breast Cancer Foundation, Canadian Breast Cancer Network, and Canadian Cancer Society. These resources mostly offer what we have termed more biomedical or *facts and figures*–type information related to breast cancer (eg, illness stages and surgery procedures) and may occasionally include some experiences of women as testimonials. After reading these resources (20 min), the participants completed a questionnaire (15 min). We considered adopting the questions from the eHealth Impact Questionnaire evaluation tool [28] for this study; however, to align the tool with the content and process used in this study, we substantially adapted the questions but retained the 5-point Likert scale. This process was then repeated for the information on the experiential information in the HERS app. The focus group ended with a discussion of the value of biomedical-oriented information resources and experiential app resources.

## Results

In this section, we present a summary of the results for each phase of the project. See Multimedia Appendix 4 for a Powerpoint presentation of the research study.

### Phase 1: Understanding Women’s Preferences and Needs Relating to the HERS App

#### Information Needs Related to Surgery

A synthesis of the analysis of the information needs of women during the surgery phase (secondary analysis and focus groups) is shown in Textbox 1.

**Women’s experiences with information related to breast cancer surgery.**

**Information received**

- Women’s contrasting experiences with regard to the information that they received was striking; some felt that they had received excellent information and others felt they were hardly informed.
- For the majority, it seemed as though there was both too much and too little information.

**Information needs**

- Women described that they were unaware of their information needs (“I think I didn’t know what I needed to know”) after finding the lump and while preparing for surgery.
- Women were understanding of the limited availability of health care professionals.
- Women described a need for experiential information on issues, such as impact on life, intimacy issues, and how to tell children.
- Women found that biomedical information about breast cancer was covered the best (in comparison with experiential or pragmatic information).
- The flow of information typically improved once patients saw their oncologist.

**Lack of information**

- Information in relation to care practices and surgery: wound care, preparation for surgery, how it will look, pain, and check-up frequency
- Experiential information: return to work, telling children, and effects on relationships. Women felt that this kind of information should be considered essential for patients.
- Women experienced a lack of information about and support for decisions related to surgery and reconstruction. They did not receive sufficient information about the importance of exercise after surgery.
- Women described feeling misinformed.

**Helpful information**

- Information from health care professionals: information session on breast cancer in hospital, visual explanations (eg, drawings showing the drains), link to a web-based decision-making tool, referrals to a sexologist and physiotherapist
- Personal resources women used to find information: websites, research articles, support groups, experiences of others, a specialized boutique that provides postmastectomy clothing, and being accompanied by a friend of a family member who can help remember the information shared during appointments with health care providers.
- Support groups: support groups were found to provide helpful resources. However, most women were only informed of the existence of a support group after surgery.

#### Value of Experiential Information

In the focus groups, women were shown some relevant experiential videos that were published on our website (Canadian Health Experiences Research Network, 2021 [26]) to discuss the value of experiential information; women described experiencing a certain comfort from watching these videos (quote 1), an ability to learn from other experiences (quote 2), and how it may have influenced their decision making (quote 3):

It’s nice to see um I would have liked that, you know. To see some real-life women, you know, like really talking about it and saying something about it. It would have been comforting, you know, because sometimes you, you just don’t know. And by the time you get into these support groups you’ve already gone through a lot of the uh stuff, the surgery....

Quote 1

So a lot of my friends were telling me “Don’t worry, radiation it’s not bad you’re going to be fine.” But I would have liked to have maybe a buddy or a nurse or somebody who I’m looking for people who have had the same thing that I have. Because I wanted to know what was your experience? Did you have the same symptoms that I had?

Quote 2

But I think before I had my surgery when I was making decisions surgery or chemo and I saw, if I saw her video it might have really influenced that decision.

Quote 3

They further said that the videos might help women who are going through a similar experience so that they feel less alone. They also highlighted the fact that women in the videos described real issues (eg, sexuality or talking to children about their diagnosis) that were not always easily discussed.

The videos also raised concerns. One participant felt that video clips may also negatively influence decision making. Another concern was related to how users were to filter and validate this type of anecdotal information. Despite the two challenges raised within the group, participants agreed that there should be a place for these kinds of videos; the participants suggested that the project team should balance more provocative videos with other videos that present solutions or different experiences of similar situations.

#### Key Design Preferences

Focus group participants were asked to describe their preferences for a mobile app and were also asked to give their opinion to certain proposed features for the app (such as the possibility to take notes); a summary of this discussion can be found in Textbox 2.

**Key design features for the Health Experiences and Real Stories app as discussed in focus groups to inform the development of the app.**

**General feedback**

- Availability of information that relates to user’s own situation
- Offer a range of experiences and information
- Important to include different age groups, stages and severities of breast cancer, marital status, and whether they have children
- Include *how to* videos
- Information should help women to normalize their experiences and include the message that each experience is unique

**Videos**

- Personal features of speakers (eg, age, ethnic background, and educational level) not defining of the information
- Provide culturally sensitive information
- Don’t overemphasize or profile certain personal attributes as videos with speakers with different characteristics may still be relevant for users with other characteristics
- Topics should be grouped according to stage and by topic including social impact
- Include information on choices for surgery and posttreatment experiences
- Include a wide variety of experiences

**Language use**

- Use language that can be understood

**Resources**

- Provide links to other resources
- Resources should be up to date and cover a wide range of information needs
- Ability to save the link.
- To increase awareness, multiple women suggested using social media outlets such as Facebook

**Note-taking**

- Could relieve some stress accompanied with getting information
- Helpful to prepare questions

### Phase 2: Content Development

More than 175 illustrative clips, with original video, audio, and text clips from the interviews with a length of about 1-2 minutes, were extracted from the recordings of the 39 interviews with women with breast cancer (n=35) and DCIS (n=4). They were related to various topics of breast cancer surgery, including preparation for surgery, types of reconstruction, and body image. These serve as the content of the HERS app.

#### Topic List: Core Content

The thematic analysis of the interview data defined the core content for the app to be organized within 11 major topics and 23 subtopics (Multimedia Appendix 2). The creation of this framework was an iterative process and was discussed with an interdisciplinary team and advisors. An additional search filter was added for the treatment phase (before surgery, surgery, after surgery, reconstruction, and impact on life). The themes and filter enable the recommender system to provide tailored information. All clips were tagged with keywords from the subthemes and treatment phase in a database server. Clips with medical information were reviewed by our health care professional advisors, such as surgeons, oncologists, and radiotherapists. Clips with potentially distressing information or unusual experiential information were reviewed by a psychologist, a representative of a breast cancer organization, or women diagnosed with breast cancer. After review, a small number of clips were removed from the database to mitigate any potential misinterpretation and an explanatory text was added other clips. The clips were then prepared and uploaded for video storage on YouTube.

#### Preparation of an Introductory Instructional Video

Members of the project team used the app *Powtoon* to develop a short plain language introduction video, which was included in the app for users to review as a guide to the HERS app.

### Phase 3: Technical Development of Mobile App, Recommender System, and Back End

**Figure 2:**
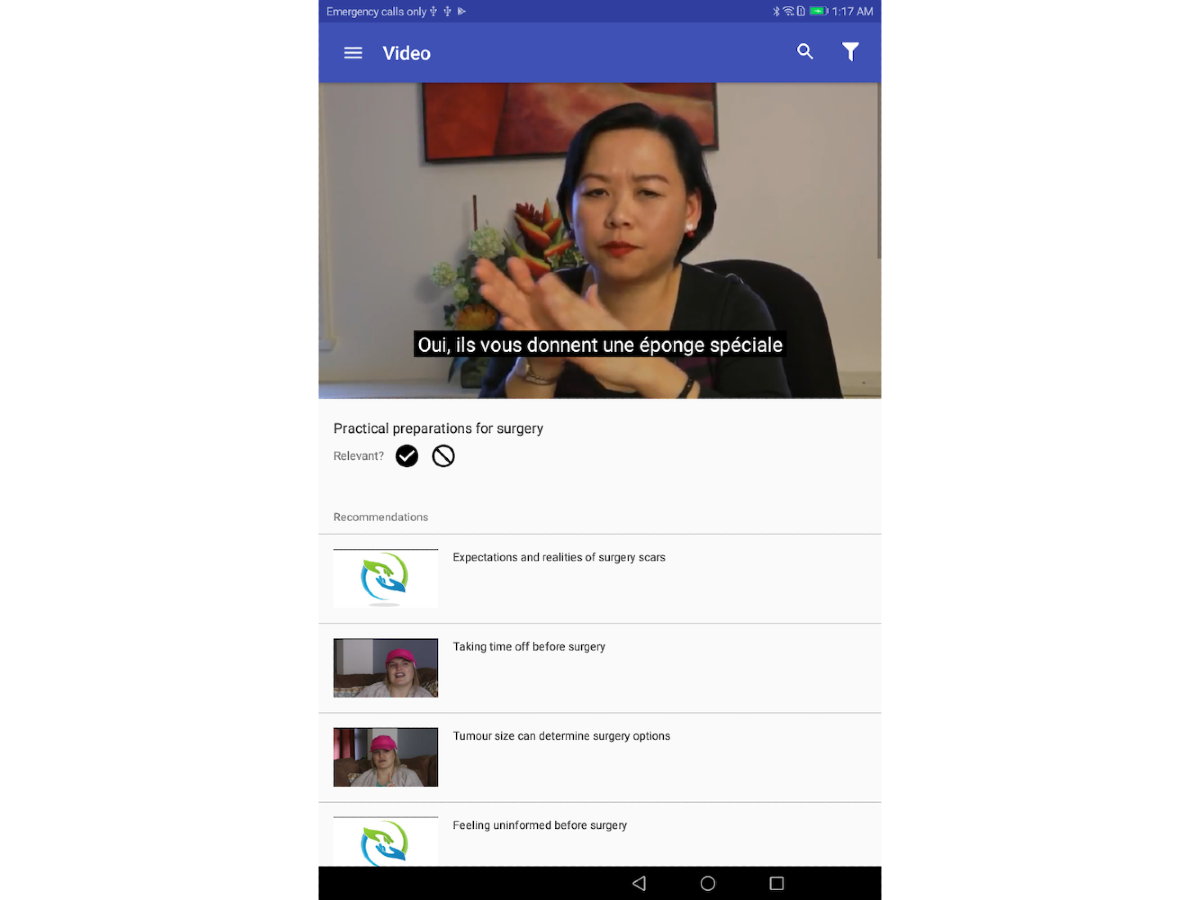
Sample screenshot of app interface.

The requirements and suggestions as described in the former two sections, together with the team’s interpretation of the requirements of the app, supported the development of the HERS app (see Figure 2 for a sample screenshot of the app interface).

Multimedia Appendix 3 details the functionality of the app. As previously described, the ability of the app to provide tailored information was a key design consideration. This was achieved through the development of a recommendation engine.

The recommendation engine performs content-based matching by recommending videos of speakers with similar characteristics as the user (based on user profiles such as age, marital status, and profession) on topics selected by the user and through collaborative filtering (videos related to videos liked by the user and by *similar* users).

The following three key principles informed our approach to recommendations:

1. Make the most informed recommendations initially
2. Increasingly reduce designer bias and let users drive recommendations
3. Account for diversity in recommendations

As expected, in a user’s very first session, little is known about what kinds of content the user is actually interested in. Nevertheless, we make informed *guesses* based on the user’s demographic information as registered in the user’s profile and match that against the speaker profile in the metadata of the videos. After at least one session, the app analyses the user’s indication of topics of interest (either done explicitly in the preferences view or implicitly by marking one or more videos as *relevant*). In addition to making recommendations based on similarity matching between users and videos, the app also makes recommendations based on diversity. The idea is to help users discover interesting content even if their preference settings and usage history do not indicate that the content is relevant to them. Heuristics are used to select videos on the diversity criteria, for example, videos that are most marked as relevant by the community.

### Phase 4: Exploring Preliminary Feedback From Potential Users

#### Feedback

The pilot app, containing a total of 175 clips extracted from interviews with 39 women, was tested by potential users (see Textbox 3 for more detailed feedback).

**Feedback on general aspects, videos, and resources of the Health Experiences and Real Stories app after testing the app.**

**General feedback**

- The app could help process information received from doctor (not all information was retained from doctor’s visits)
- The app could be so interesting that the user would stay too long—guide users to access videos step by step
- The app looked professional, was well organized, and was easy to navigate. Some technical problems with the app—in some instances, the app would freeze
- The tool could help women be better prepared for the next stage

**Videos**

- Liked speakers’ personalities
- Presentation and tone were described as intelligent and nonpatronizing
- Appreciation of range of topics and realistic videos
- Liked the ability to choose own videos or skip videos
- Length was good (not too long)
- If videos felt short, participant liked that other videos were available or that they could replay videos
- Organization by topic was helpful
- One participant liked the *snippets* of information; it worked for her
- Information well balanced: not too happy nor too sad
- One participant described how she recognized the experiences of one speaker

**Resources**

- Resources were appreciated and perceived as trustworthy
- Good step-by-step process
- Professional outlook and quality of resources
- Information would have a relaxing and calming effect on women during diagnoses phase
- Add contact information and feature for asking questions
- Appreciation of combination of biomedical and experiential information

#### Survey Results

In total, 5 female participants were asked to complete the questionnaire twice: first after reading selected biomedical information resources that can be found on reliable websites and then after visiting the experiential section of the HERS information app (see Table 1 for the results). The results revealed that participants positively perceived the biomedical information, and the results were slightly better after viewing the app information, for example, they seemed to feel less overwhelmed with information. However, this is a very small sample, and further testing is required.

**Table 1:** Results from survey questions (N=5)^a^.

Question	After reading biomedical information^b^, median (range)	After reading app information^c^, median (range)
1. I feel better informed after viewing the resources.	4 (3-5)	4 (4-5)
2.I had a lot of my questions answered by the resources or this app.	4 (3-5)	4 (4-4)
3.I better understand the information I need to prepare for the next steps in treatment or stages of the illness.	4 (3-4)	4 (4-4)
4.I was able to easily find the information I was looking for.	4 (3-5)	4 (4-5)
5.The resources made me feel less lonely or isolated.	4 (3-5)	4 (4-5)
6.If a friend or family member were in a similar position, would you recommend the resources or this app to her?	5 (3-5)	5 (4-5)
7. I have a better understanding of where to seek information about breast cancer and surgery.	4 (4-5)	4 (3-5)
8. Looking at the resources or site raised more questions for me than it answered.	3 (2-4)	2 (2-3)
9. There was so much information here, I felt overwhelmed.	3 (1-4)	2 (1-4)
10. It was difficult for me to relate to the information or the stories in this resource.	1 (1-3)	2 (1-2)
11. The information on this app will not help me feel more confident discussing my questions and concerns with my doctor(s).	1 (1-2)	1 (1-2)

^a^The questionnaire used a Likert scale of 1 to 5 for all questions except question 8 (1=strongly disagree; 2=disagree; 3=neither agree nor disagree; 4=agree; 5=strongly agree). For question 8, the responses represented the following: 1=definitely would not; 2=would not; 3=neither would nor would not; 4=would; and 5=definitely would.

^b^Total criteria satisfied (rated 4 or 5 for question 1 or 7 and rated 1 or 2 for question 8 or 11) was 9.

^c^Total criteria satisfied (rated 4 or 5 for question 1 or 7 and rated 1 or 2 for question 8 or 11) was 11.

## Discussion

### Principal Findings

This paper describes the development and piloting of a mobile app that provides tailored information for women, based on their preferences and needs, about others’ experiences of breast surgery. This system draws upon an existing evidence-based data set of video narratives featuring Canadian women’s experiences of breast surgery and uses advanced computing engineering and machine learning in the design of a recommendation algorithm to provide tailored information (similar to the popular Netflix platform). Our aim was to contribute to efforts that provide the right information at the right time and in the right format to help women manage information at a critical time in their care journey for breast cancer. In a preliminary pilot test, we found generally positive responses to questions regarding the content and value of this type of e-tool.

Our findings regarding women’s information needs and their experiences related to missing relevant treatment information and information overload are consistent with the existing literature [29-31]. There is a need for the development and assessment of more interventions that help information seekers manage health information overload [3] and a need to develop information filters to help information seekers identify relevant web-based health information [5]. None of the identified articles in a recent systematic review on mobile apps for breast cancer care used a recommender system to generate tailored information and only three mentioned a feature with regard to tailoring information [12,13]. Although the use of recommender systems is still sparse in the health sector, it has the potential to contribute to tailored health interventions [32], and mobile apps for breast cancer could contribute to reducing information overload by offering tailored information and machine learning. This study contributes to the scarce existing knowledge related to evidence-based eHealth apps that are designed to provide information and support for women with breast cancer in addition to the novel application of a recommender system to do so. Scientific literature presenting narrative-based apps designed on the basis of rigorous research is virtually nonexistent, as per our preliminary review of the literature.

### Considerations for Future Research

Research in the last decade has demonstrated the challenges of measuring the health effects associated with health information usage of information offered on the web or through web-based tools. These mechanisms are complex and interrelated with many factors. Tools, such as the one described in this paper, offer the potential to contribute to new knowledge and to a better understanding of information-seeking practices because of the ability to relate the user feedback on the videos (relevant or not) to the app usage as well as to the personal characteristics of the user, the speaker, and the content of the video. For example, the app makes it possible to better understand whether certain personal user characteristics, such as age, ethnic background, having children, marital status, and literacy level, influence one’s preference for certain speakers with similar characteristics. In the future, it may also be possible to add other important characteristics that influence information needs [29], such as preexisting knowledge of the illness and preference for more basic or advanced illness information. A recently developed narrative taxonomy defines three different kinds of narratives: process, experience, and outcome narratives [33]. The authors argue that, for each type of app (information tool, decision aid, and behavior change information), different narrative types should be used. For example, for an information tool, creators should include narratives about a process or experience but exclude narratives focused on the outcome. Future research should aim to better understand the impact of different types of narratives on the effectiveness and uptake of particular mobile apps for health. Finally, it is important to further test the impact of the app on the users, for example, through a randomized controlled trial.

Our research group is part of a global network of researchers who conduct similar research in their respective countries. This app offers the possibility of expansion by including clips from international collections of breast cancer narratives or by creating similar tools that use scientifically gathered, personal experiences of a variety of health conditions. An international working group is currently formed with member countries of an international network for patients’ experiences [34] for the application of the HERS app elsewhere.

In addition, it is important that the app also offers information that the user does not yet know that she needs to know (eg, information on lymphedema exercises and the need to consider fertility treatment). Research on how women can best be introduced to these subjects through the app would be important. For instance, should the recommender system continuously offer clips on these specific subjects or should women be made aware of them through other pathways? It would also be important to consider how other unexpected events or complications during treatment (such as drain tube removal or unclear margins) should be introduced without engendering unnecessary fear or anxiety.

### Limitations

The limitations of this study are primarily related to the scale and the limited nature of the pilot study. We convened a small sample of breast cancer survivors to conduct a preliminary *test* of the functionality and user experience of the app. Admittedly, our targeted app users are women who are yet to undergo surgery. In this study, we chose to involve women who had already undergone surgery to provide retrospective insights into how this app would have helped them during their journey. The participants first accessed biomedical information and then experiential information; the order of this information may have influenced their responses to the survey. This would need to be explored in future trials of this product. Although the results were not intended to provide any statistical significance, we were encouraged by the favorable trends in their responses. We will now seek additional opportunities to expand the evaluation of the app to involve more women with breast cancer who are yet to undergo surgery, develop an iOS version of the app for Apple mobile devices, invite other health experience research groups using similar methods to test the addition of an expanded data set (video narratives gathered in other jurisdictions and on different health conditions), optimize the saturation of information within topics identified for this app, and consider a cloud-based deployment to eliminate the need to be bound to a specific server. The aim of our next phase of development will be to formally evaluate the utilization and impact of this eHealth tool on women’s shared decision making and the perceived needs for information and support around the time of surgery in their breast cancer journey.

### Conclusions

In this paper, we provide preliminary evidence for the feasibility and acceptability of an innovative eHealth app designed to tailor experiential information for women preparing for breast cancer surgery. The HERS app, based on a recommender system, is a unique attempt to ensure that women receive the right information at the right time in the right format; however, further testing is still required to measure the impact of the app.

We believe that these kinds of tools offer great potential to improve health information competence and reduce information overload, while ensuring that women receive timely, relevant information that meets their needs, and they complement more factual, biomedical information about their illness that they receive from their care team and other sources.

## Appendix

Multimedia Appendix 1 System structure of breast cancer video service.

Multimedia Appendix 2 Framework for coding and Health Experiences and Real Stories app.

Multimedia Appendix 3 Description of app functions.

Multimedia Appendix 4 Bringing Netflix technology to video narratives of experiences of breast surgery: helping women navigate the information tsunami.

## Abbreviations

DCIS: ductal carcinoma in situ
HERS: Health Experiences and Real Stories
SMHC: Saint Mary’s Hospital Center
