# Supplementary material for: Using a Mobile App–Based Video Recommender System of Patient Narratives to Prepare Women for Breast Cancer Surgery: Development and Usability Study Informed by Qualitative Data
Source: JMIR Form Res. 2021 Jun 2;5(6):e22970. doi: 10.2196/22970 (PMC8209533; doi:10.2196/22970)
Supplement: Multimedia Appendix 1 [file formative_v5i6e22970_app1.docx]

*Mulitmedia Appendix 1. System structure of breast cancer video service*

**
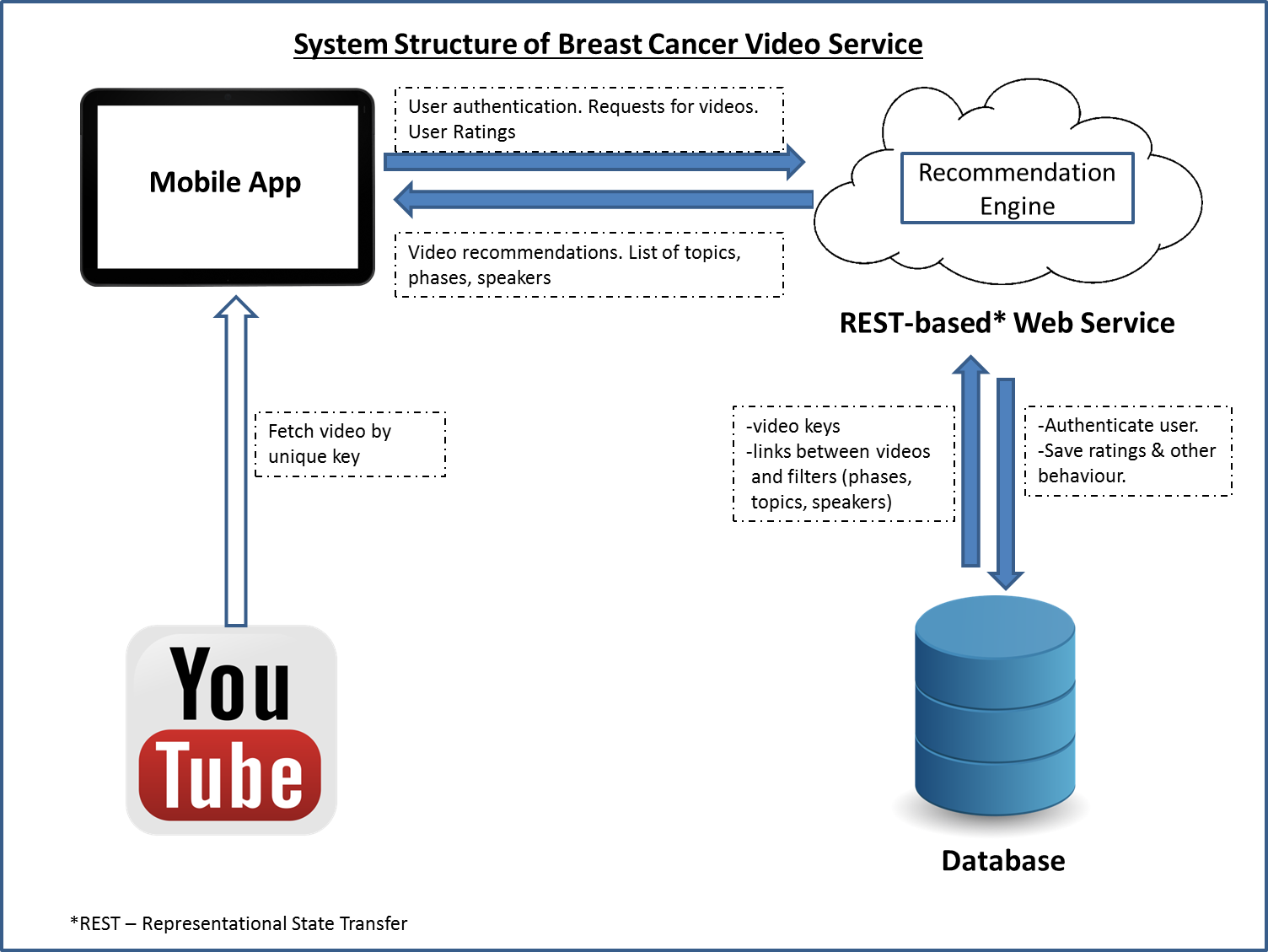
**

*Explanatory Notes for Figure 2:*

- 1. **Mobile Application Client (aka Mobile App):** A piece of software that runs on the mobile device (phone or tablet). It constitutes the interface for watching videos, setting filters/preferences, etc. As seen in figure 1 below, the mobile app communicates directly with only the web service and YouTube. See the end of this report for a detailed list of functionalities of the mobile application and actual screenshots of the app. (For instance, user registration, user login, video viewing and rating – relevant or not, recommendations provided by app, search functionality, browse function by “speakers”, browse function by topic, profile information, speaker preferences, links to reliable resources, language option (French/English), note taking and feedback button).
  2. **REST-based Web Service:** We developed a web service based on the principles of representational state transfer. This is a piece of software that provides the backend logic for all the functionalities of the mobile app. The web service communicates with the database to retrieve, process and store information from the mobile app. The web service was deployed to the Microsoft Azure cloud platform for user testing.
  3. **Recommender System:** The recommender system is another software developed for suggesting videos to users based on their profile and (implicit and explicit) preferences. It is triggered by a message passing algorithm to analyse data in the database about a specific subject and save appropriate recommendations which are then picked up by the web service for onwards submission to the user via the mobile app. The recommender system also runs on Azure presently. This will be transferred to a hospital-based Azure account in the Fall 2018.
  4. **Database Server:** Metadata about speakers, videos and users of the app are stored in the database. Examples of speaker data in the database are name, age, and type of breast cancer. Examples of video data in the database include characteristics of the speaker in the video and the topic of the video. The metadata stored in the database is the key input to the recommender system. The database was developed on Microsoft SQL Server and is also deployed to Azure presently.
  5. **Video Storage (YouTube):** Selected video clips of the qualitative interviews were uploaded and stored on the YouTube video storage platform. Bilingual subtitles were also made and uploaded to YouTube channel. The mobile app, upon retrieving a video ID from the database through the web service, goes to YouTube to retrieve the actual videos.
