# Supplementary material for: Using a Mobile App–Based Video Recommender System of Patient Narratives to Prepare Women for Breast Cancer Surgery: Development and Usability Study Informed by Qualitative Data
Source: JMIR Form Res. 2021 Jun 2;5(6):e22970. doi: 10.2196/22970 (PMC8209533; doi:10.2196/22970)
Supplement: Multimedia Appendix 2 [file formative_v5i6e22970_app2.docx]

*Multimedia Appendix 2. Framework for coding and HERS app*

| **Topics and Sub-Topics** | **Filter: Treatment Phase** |
| --- | --- |
|  |  |
| **About Surgery** |  |
| Preparing for surgery | *Before Surgery* |
| **Surgery Options** |  |
| Lumpectomy vs mastectomy | *Before Surgery* |
| Single vs double mastectomy | *Before Surgery* |
| **Lymph Nodes** |  |
| Lymph nodes (sentinel node biopsy) | *Surgery* |
| **Lymphedema** |  |
| Treating lymphedema | *After Surgery* |
| **Lumpectomy** |  |
| Preparing for a lumpectomy | *Before Surgery* |
| Clear/unclear margins | *Surgery* |
| Lumpectomy results | *After Surgery* |
| Recovering from a lumpectomy | *After Surgery* |
| **Mastectomy** |  |
| Preparing for mastectomy | *Before Surgery* |
| Mastectomy results | *After Surgery* |
| Recovering from mastectomy | *After Surgery* |
| **Drains** |  |
| Recovering from mastectomy | *After Surgery* |
| **Reconstruction** |  |
| Types of reconstruction | *Reconstruction* |
| Creating nipples and areola | *Reconstruction* |
| Considerations around reconstruction | *Reconstruction* |
| No reconstruction | *Reconstruction* |
| Relationship with surgeons | *Reconstruction* |
| **Prosthesis** |  |
| Prosthesis | *Reconstruction* |
| **Impact on life** |  |
| Body image | *Impact on Life* |
| Sexuality | *Impact on Life* |
| Emotions | *Impact on Life* |
| Children | *Impact on Life* |
| **Advice for others** |  |
