# Supplementary material for: Using a Mobile App–Based Video Recommender System of Patient Narratives to Prepare Women for Breast Cancer Surgery: Development and Usability Study Informed by Qualitative Data
Source: JMIR Form Res. 2021 Jun 2;5(6):e22970. doi: 10.2196/22970 (PMC8209533; doi:10.2196/22970)
Supplement: Multimedia Appendix 3 [file formative_v5i6e22970_app3.docx]

*Multimedia Appendix 3. Description of App Functions*

| **Function** | **Description** |
| --- | --- |
|  |  |
| **User Registration** | Enables users sign up to use the app. |
| **User Login** | Enables users login to access the app. |
| **Video Viewing** | Enables users watch and rate a video. |
| **Recommendation** | Computes and displays a list of recommended videos to user. |
| **Search Panel** | Enables users search for a video by entering keywords. |
| **Database Browsing** | Enables users browse through videos in the database by filtering by speaker, phase or topic. |
| **User Profile** | Enables users to supply demographic and illness-related information used by the recommendation system in suggesting video clips. |
| **User Preference** | Enables users select speakers, phase or topic of interest. Recommender system uses this to tailor recommendations. |
| **Resources** | Provides users with links to other useful online resources which has been verified by experts. |
| **“About” View** | Provides information about all organizations that have collaborated to make this project successful. |
| **Language Options** | Provides the ability to change the language of the app from English to French and vice-versa. It also enables subtitles in the language other than that of the speaker in the video. |
