# Supplementary material for: Using a Mobile App–Based Video Recommender System of Patient Narratives to Prepare Women for Breast Cancer Surgery: Development and Usability Study Informed by Qualitative Data
Source: JMIR Form Res. 2021 Jun 2;5(6):e22970. doi: 10.2196/22970 (PMC8209533; doi:10.2196/22970)
Supplement: Multimedia Appendix 4 [file formative_v5i6e22970_app4.pptx]

## Slide 1
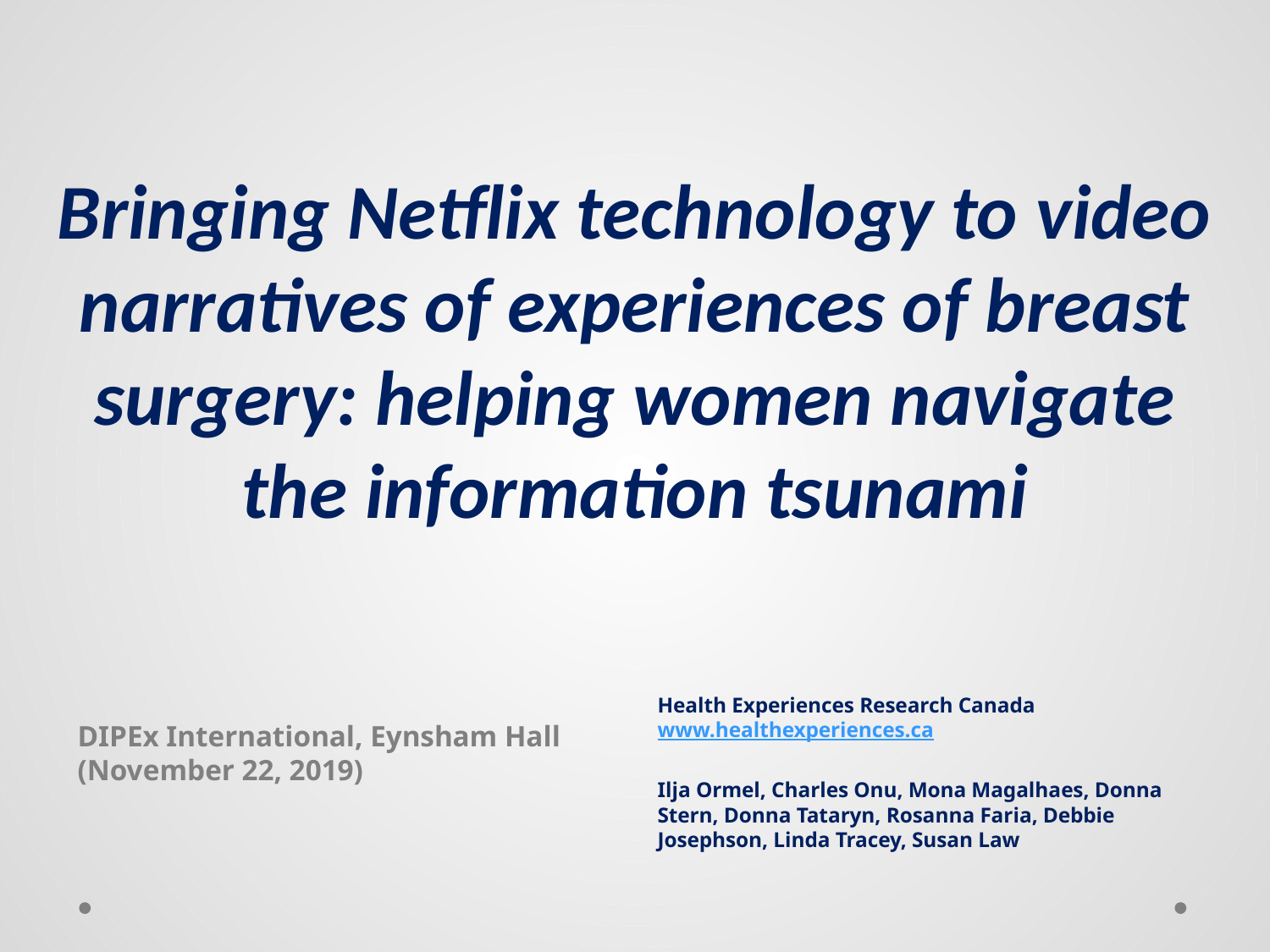

# Bringing Netflix technology to video narratives of experiences of breast surgery: helping women navigate the information tsunami
Health Experiences Research Canada www.healthexperiences.ca
Ilja Ormel, Charles Onu, Mona Magalhaes, Donna Stern, Donna Tataryn, Rosanna Faria, Debbie Josephson, Linda Tracey, Susan Law
DIPEx International, Eynsham Hall (November 22, 2019)

## Slide 2
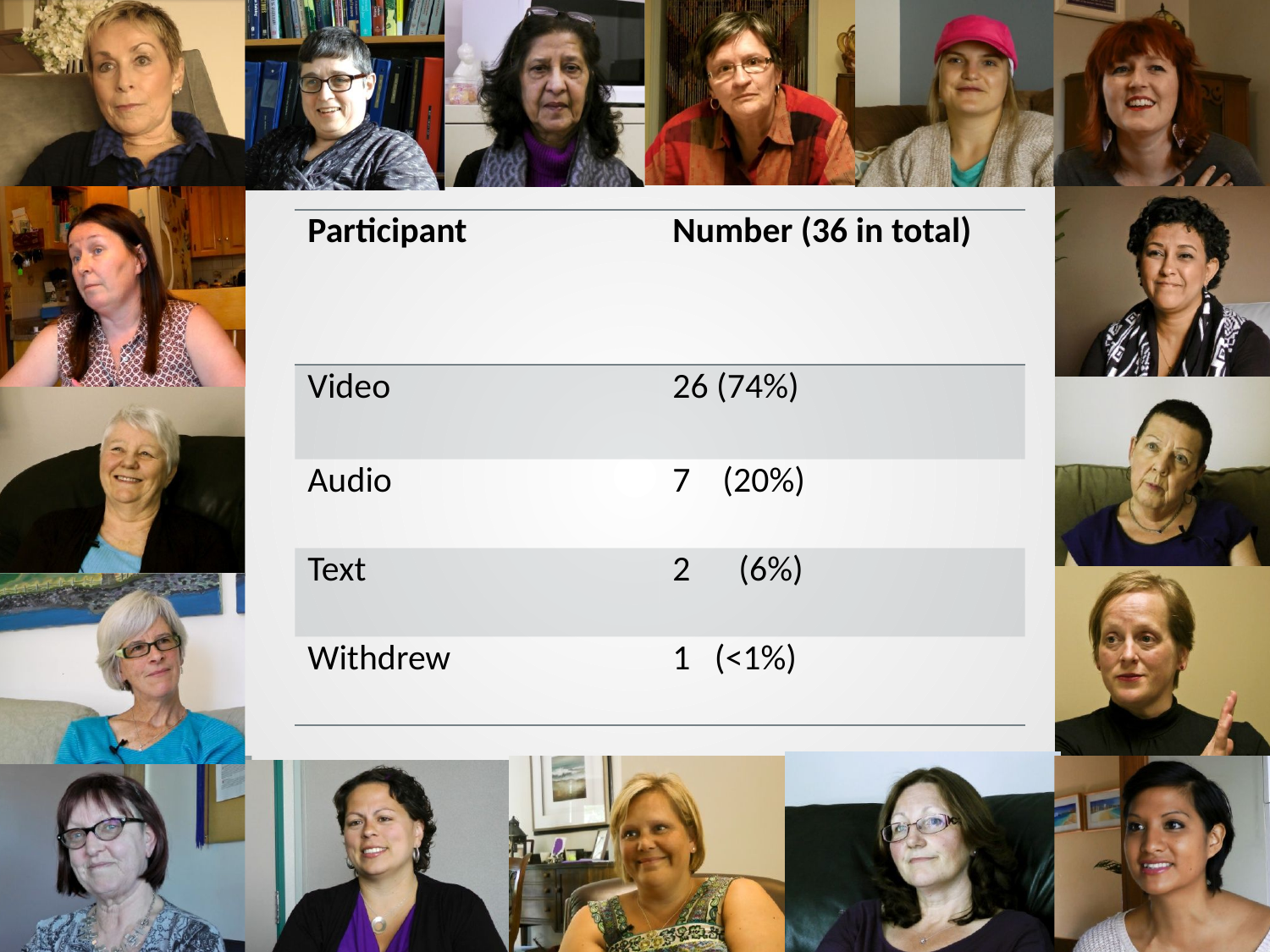

| Participant | Number (36 in total) |
| --- | --- |
| Video | 26 (74%) |
| Audio | 7 (20%) |
| Text | 2 (6%) |
| Withdrew | 1 (<1%) |

## Slide 3
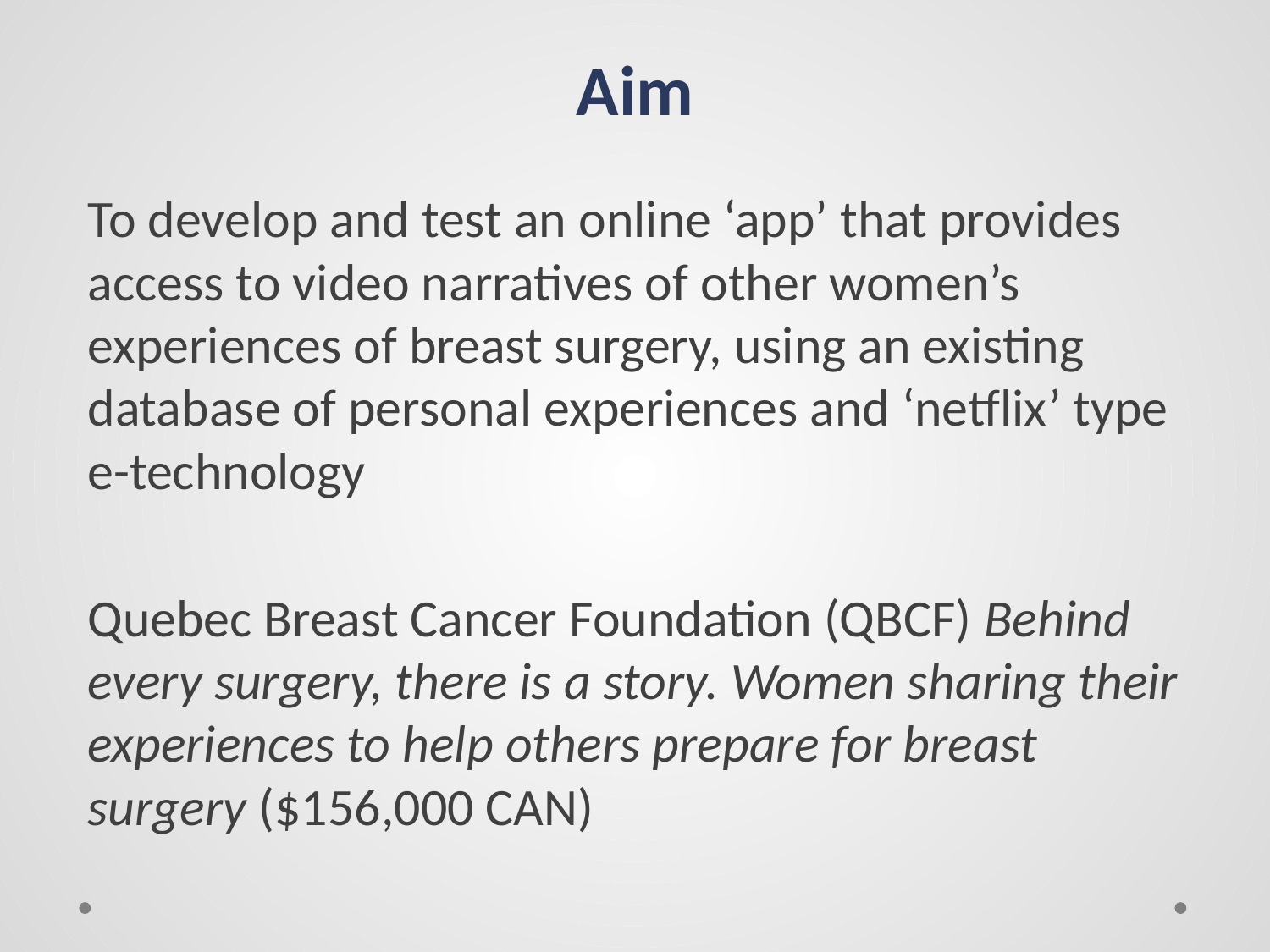

# Aim
To develop and test an online ‘app’ that provides access to video narratives of other women’s experiences of breast surgery, using an existing database of personal experiences and ‘netflix’ type e-technology
Quebec Breast Cancer Foundation (QBCF) Behind every surgery, there is a story. Women sharing their experiences to help others prepare for breast surgery ($156,000 CAN)

## Slide 4
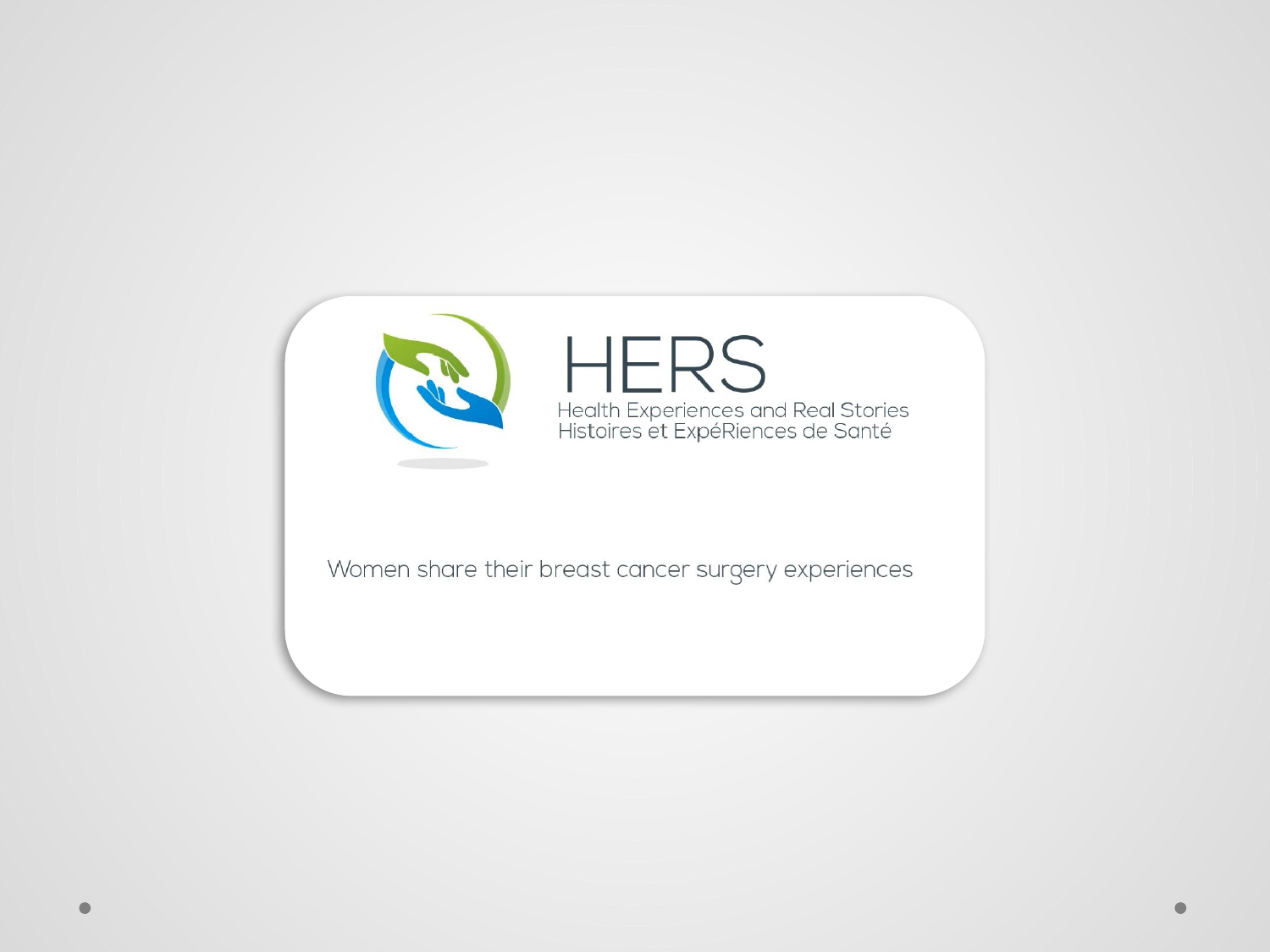

#

## Slide 5
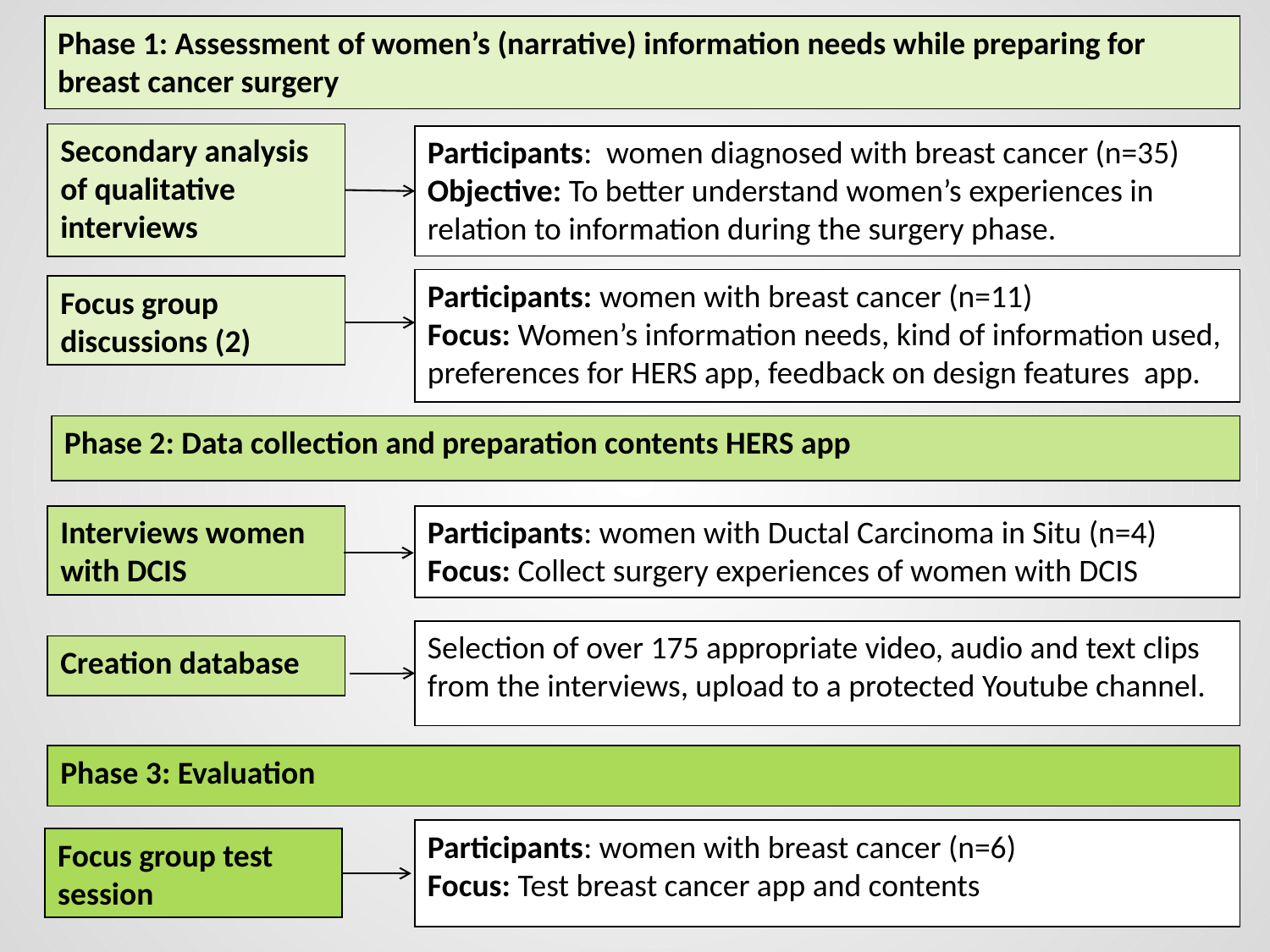

Phase 1: Assessment of women’s (narrative) information needs while preparing for breast cancer surgery
Secondary analysis of qualitative interviews
Participants: women diagnosed with breast cancer (n=35)
Objective: To better understand women’s experiences in relation to information during the surgery phase.
Participants: women with breast cancer (n=11)
Focus: Women’s information needs, kind of information used, preferences for HERS app, feedback on design features app.
Focus group discussions (2)
Phase 2: Data collection and preparation contents HERS app
Interviews women with DCIS
Participants: women with Ductal Carcinoma in Situ (n=4)
Focus: Collect surgery experiences of women with DCIS
Selection of over 175 appropriate video, audio and text clips from the interviews, upload to a protected Youtube channel.
Creation database
Phase 3: Evaluation
Participants: women with breast cancer (n=6)
Focus: Test breast cancer app and contents
Focus group test session

## Slide 6
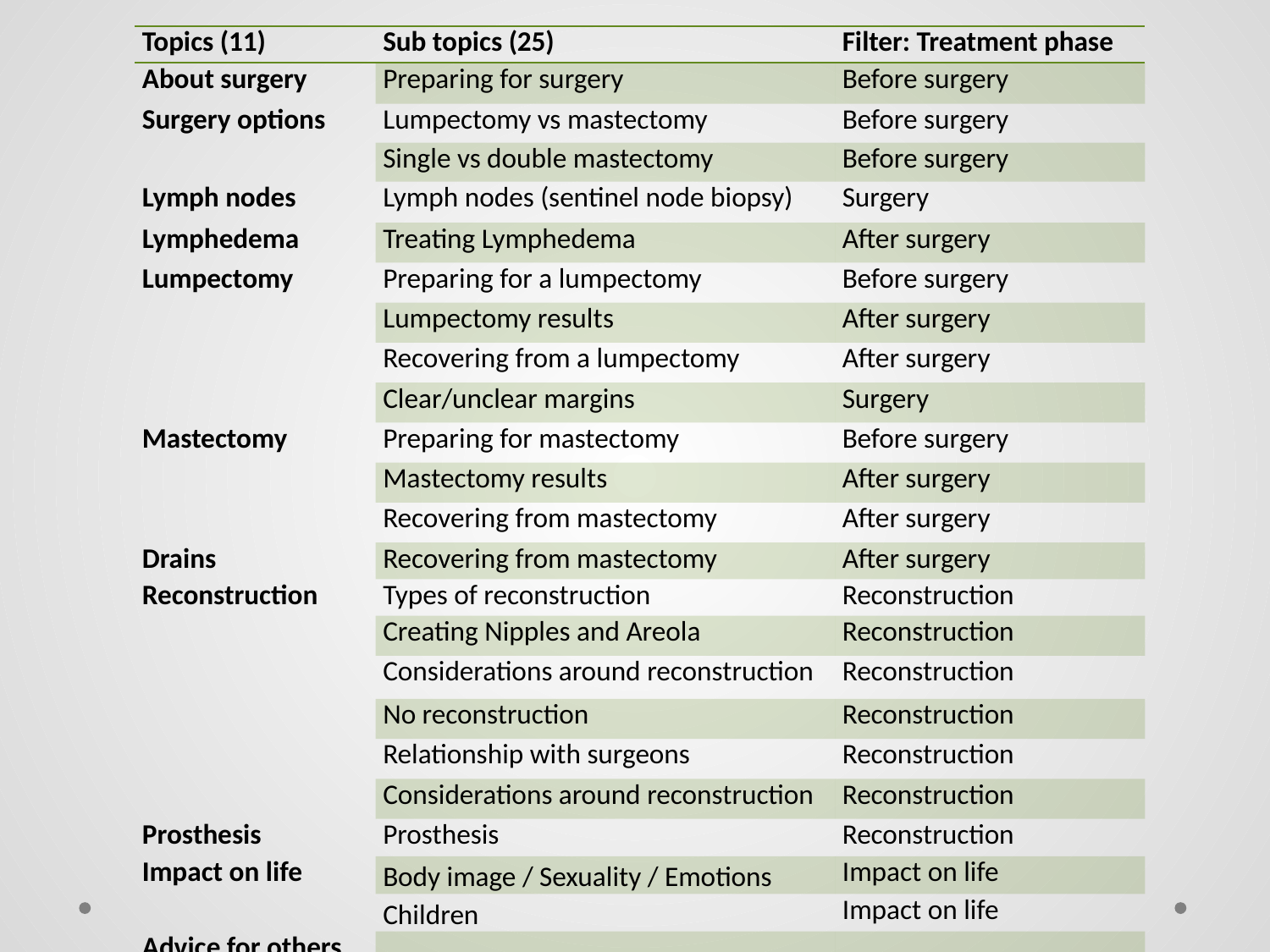

| Topics (11) | Sub topics (25) | Filter: Treatment phase |
| --- | --- | --- |
| About surgery | Preparing for surgery | Before surgery |
| Surgery options | Lumpectomy vs mastectomy | Before surgery |
| | Single vs double mastectomy | Before surgery |
| Lymph nodes | Lymph nodes (sentinel node biopsy) | Surgery |
| Lymphedema | Treating Lymphedema | After surgery |
| Lumpectomy | Preparing for a lumpectomy | Before surgery |
| | Lumpectomy results | After surgery |
| | Recovering from a lumpectomy | After surgery |
| | Clear/unclear margins | Surgery |
| Mastectomy | Preparing for mastectomy | Before surgery |
| | Mastectomy results | After surgery |
| | Recovering from mastectomy | After surgery |
| Drains | Recovering from mastectomy | After surgery |
| Reconstruction | Types of reconstruction | Reconstruction |
| | Creating Nipples and Areola | Reconstruction |
| | Considerations around reconstruction | Reconstruction |
| | No reconstruction | Reconstruction |
| | Relationship with surgeons | Reconstruction |
| | Considerations around reconstruction | Reconstruction |
| Prosthesis | Prosthesis | Reconstruction |
| Impact on life | Body image / Sexuality / Emotions | Impact on life |
| | Children | Impact on life |
| Advice for others | | |

## Slide 7
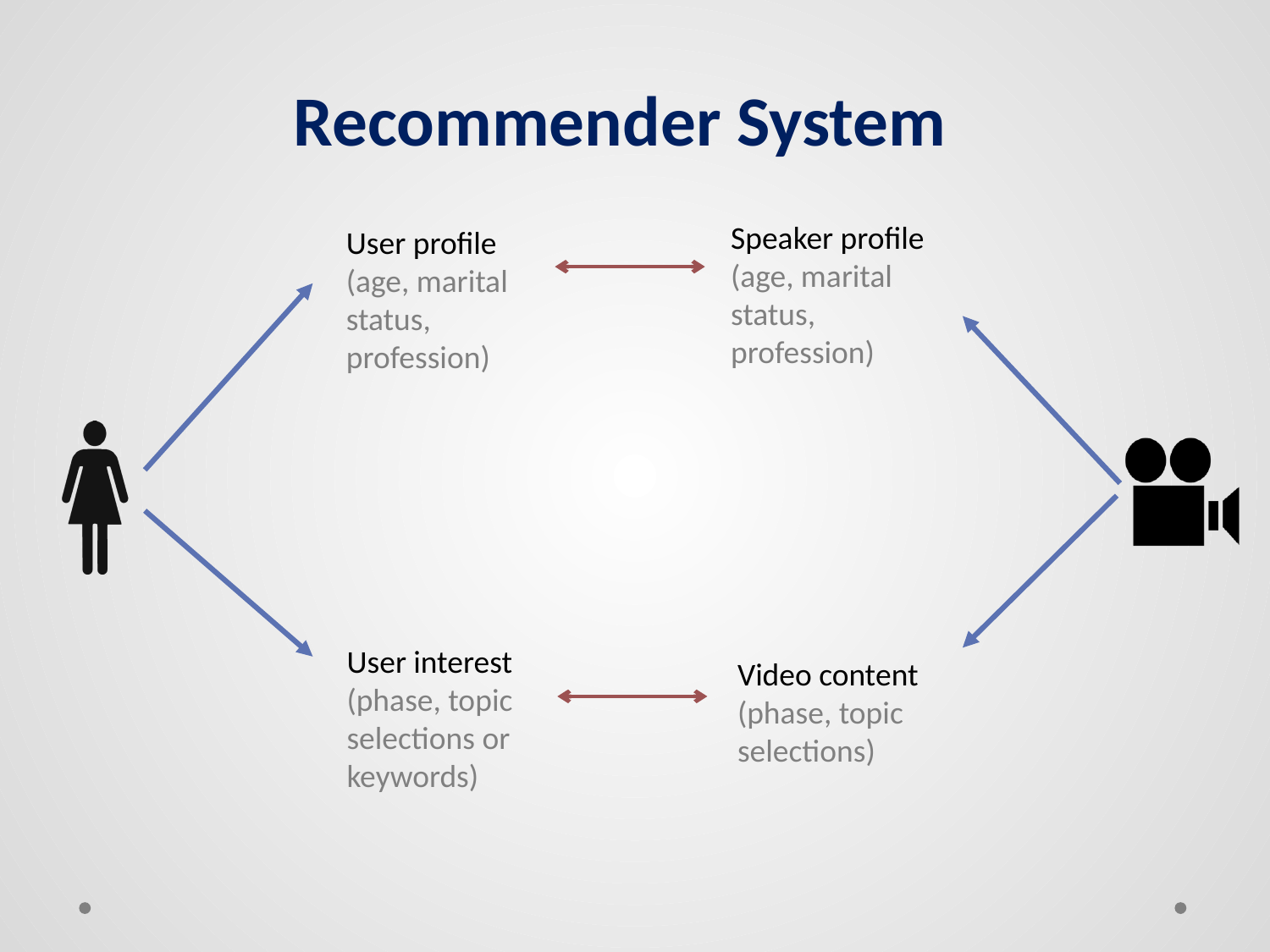

Recommender System
Speaker profile
(age, marital status, profession)
User profile
(age, marital status, profession)
Video content
(phase, topic selections)
User interest
(phase, topic selections or keywords)

## Slide 8
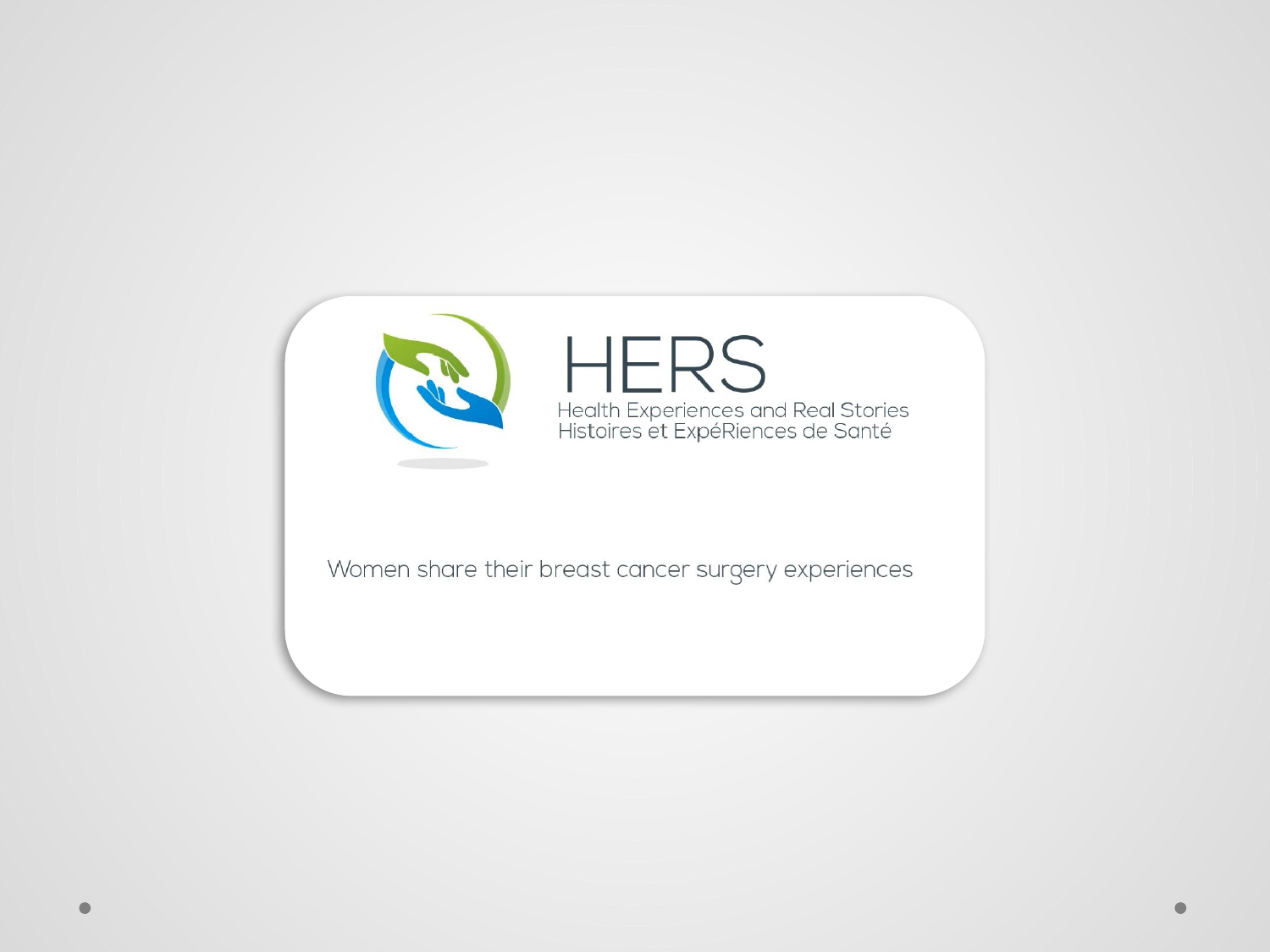

#

## Slide 9
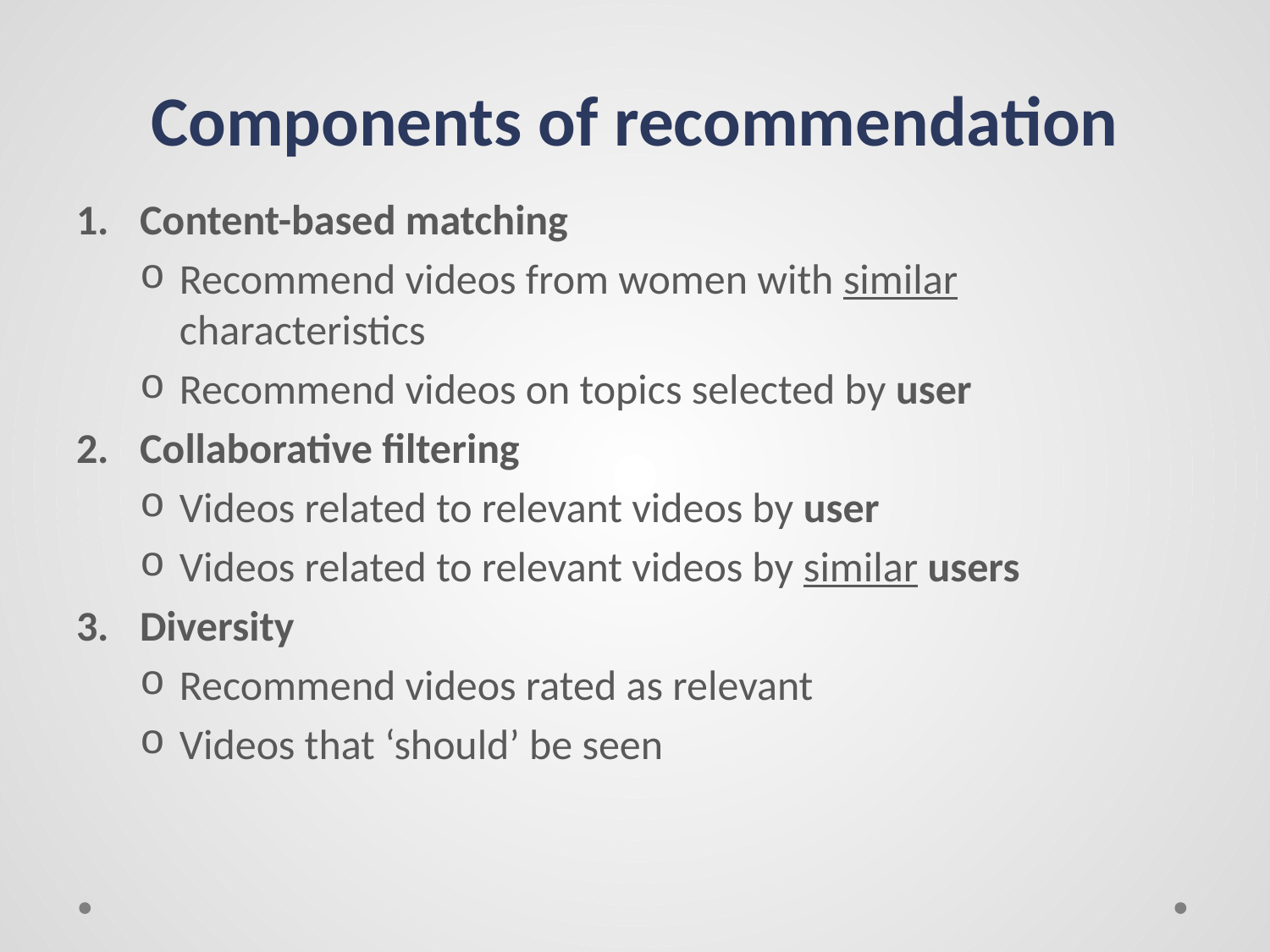

# Components of recommendation
Content-based matching
Recommend videos from women with similar characteristics
Recommend videos on topics selected by user
Collaborative filtering
Videos related to relevant videos by user
Videos related to relevant videos by similar users
Diversity
Recommend videos rated as relevant
Videos that ‘should’ be seen

## Slide 10
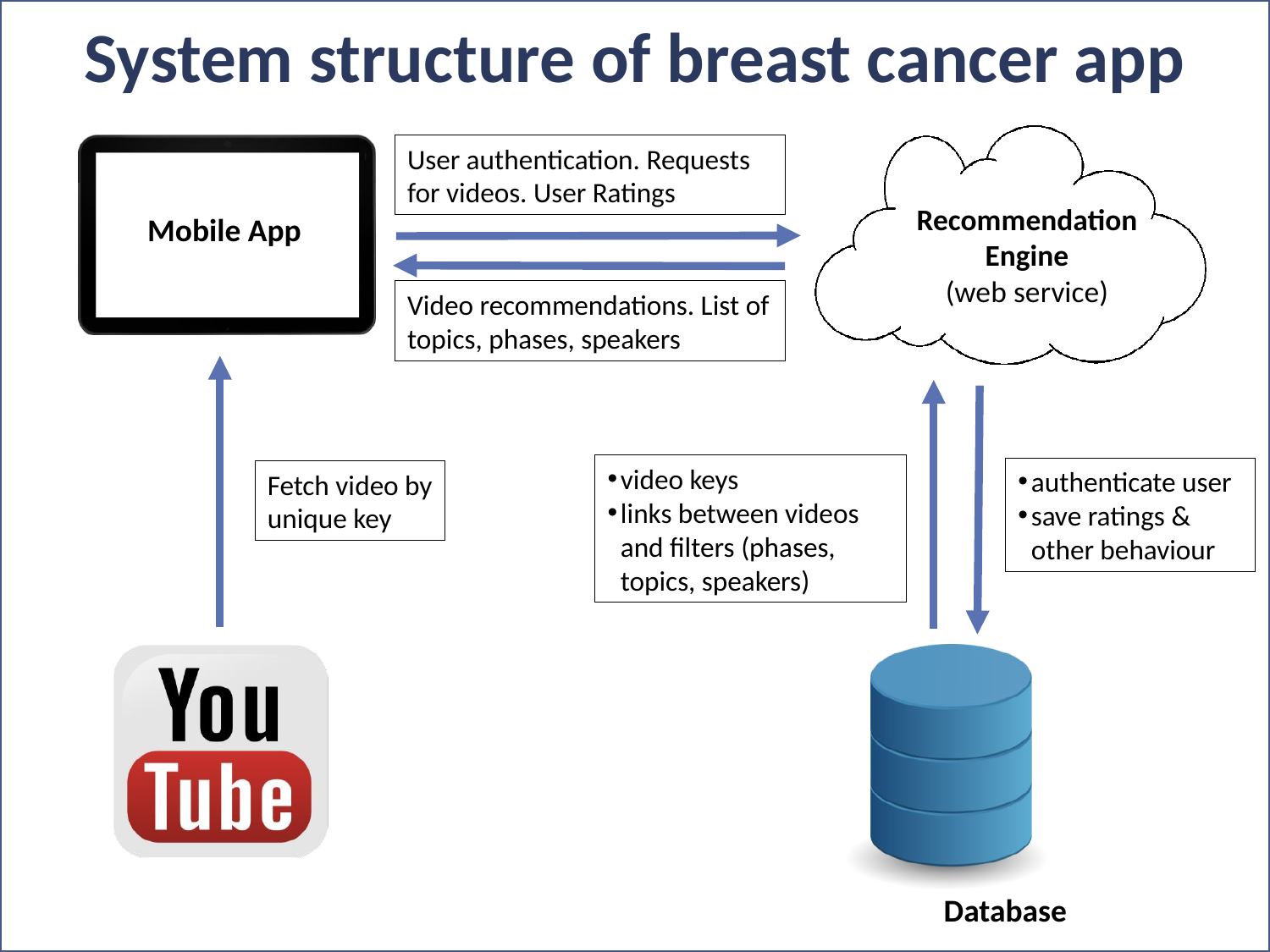

System structure of breast cancer app
User authentication. Requests for videos. User Ratings
Recommendation Engine
(web service)
Mobile App
Video recommendations. List of topics, phases, speakers
video keys
links between videos and filters (phases, topics, speakers)
authenticate user
save ratings & other behaviour
Fetch video by unique key
Database

## Slide 11
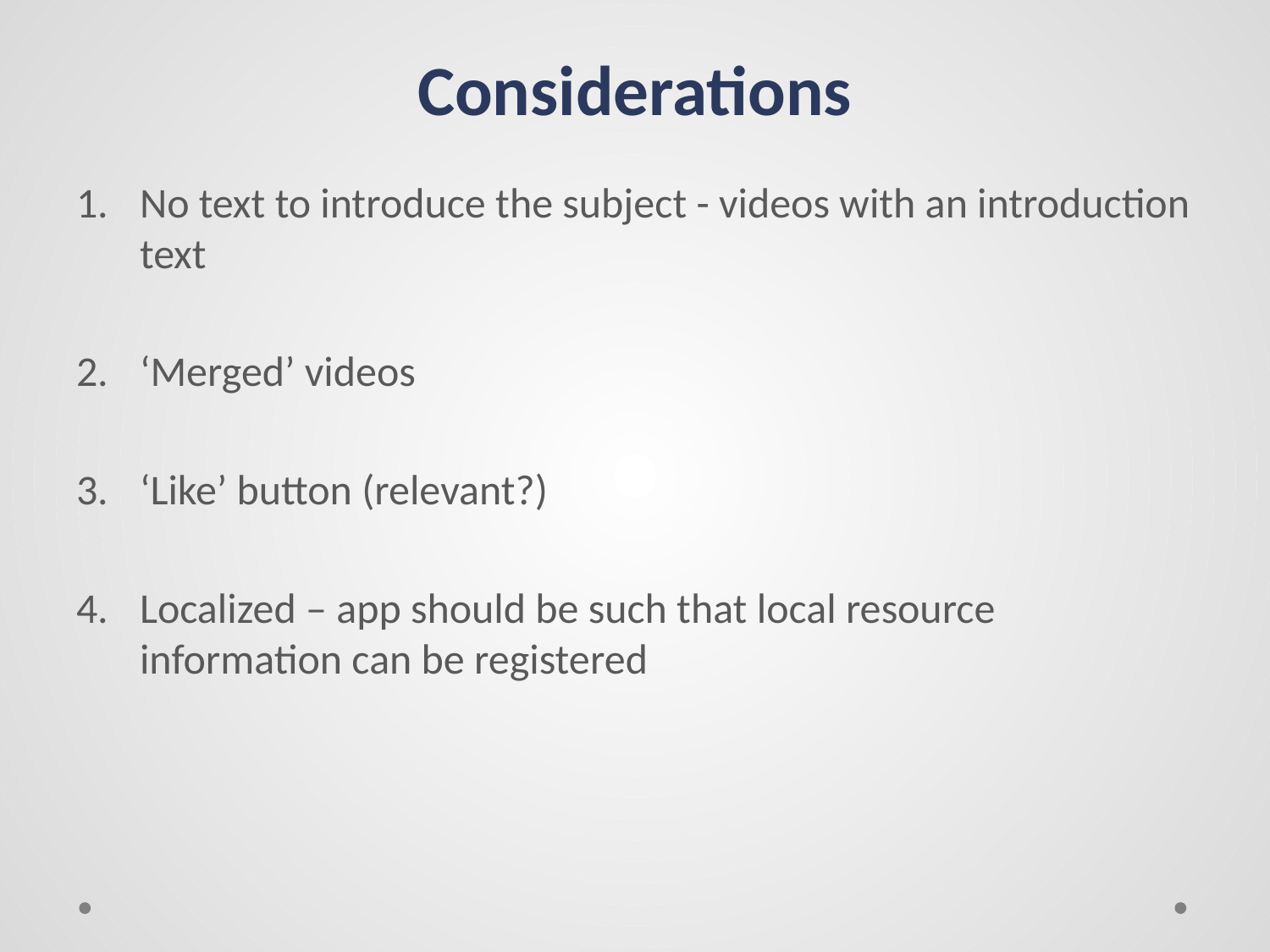

# Considerations
No text to introduce the subject - videos with an introduction text
‘Merged’ videos
‘Like’ button (relevant?)
Localized – app should be such that local resource information can be registered

## Slide 12
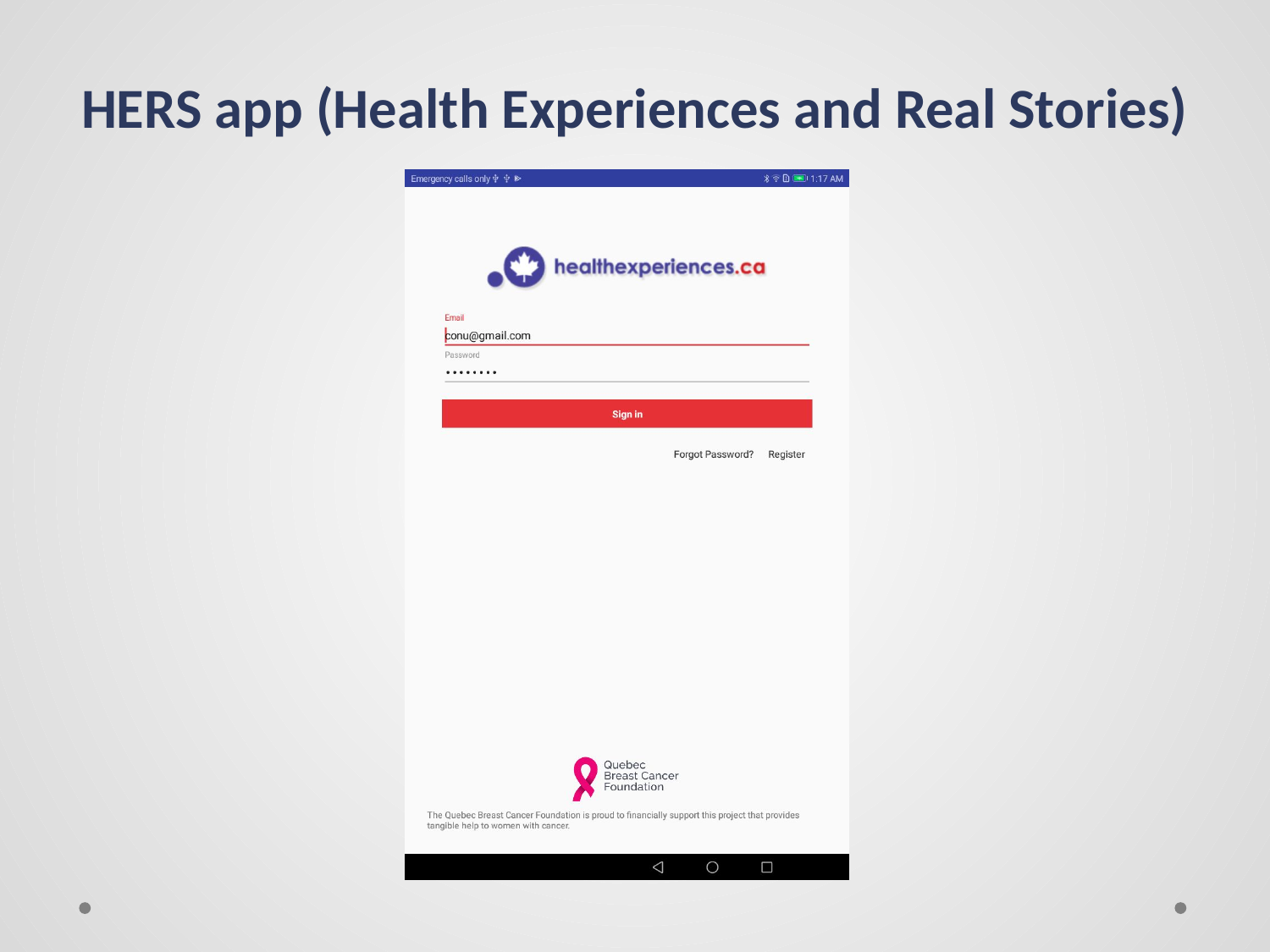

# HERS app (Health Experiences and Real Stories)

## Slide 13
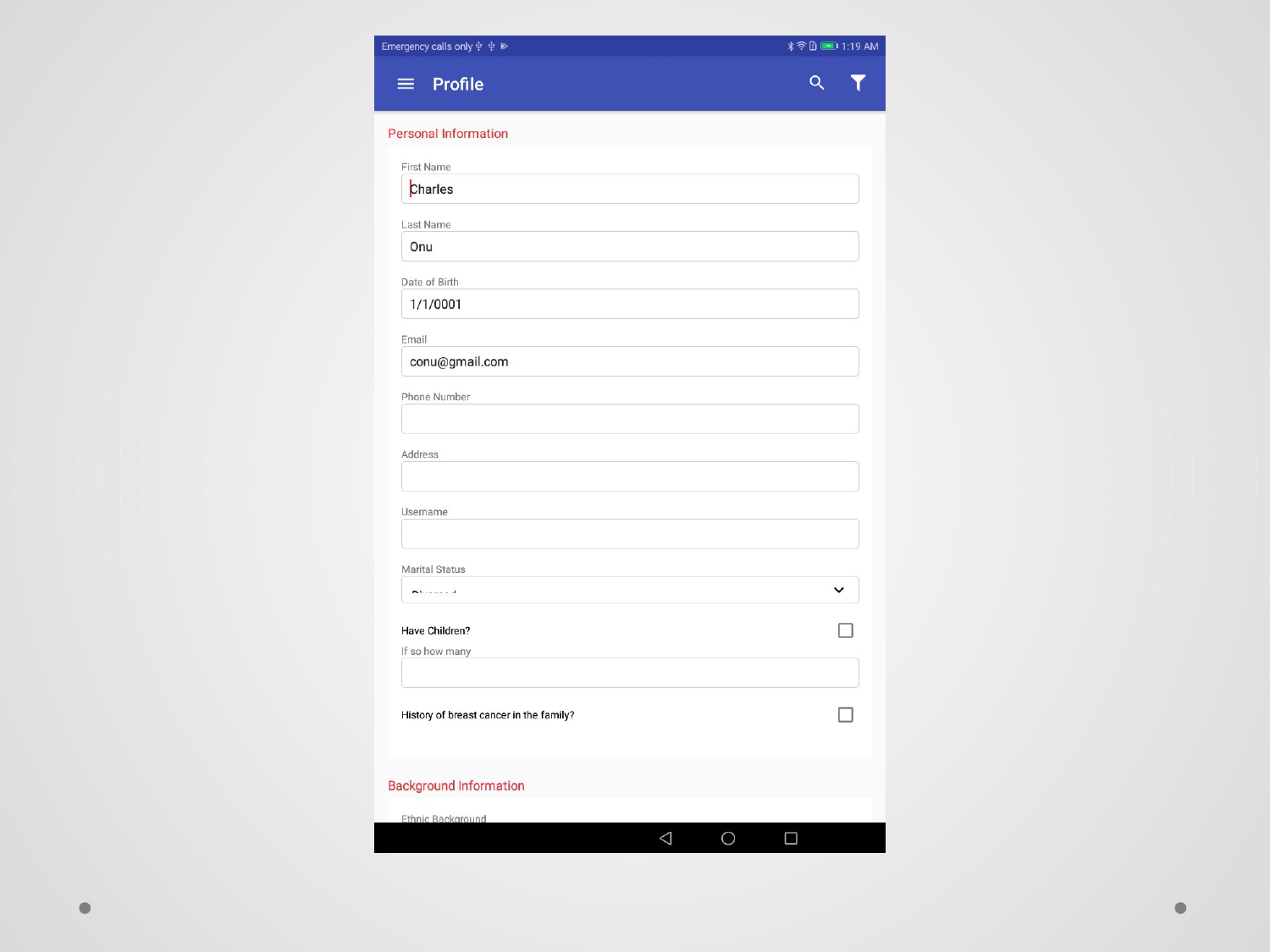

## Slide 14
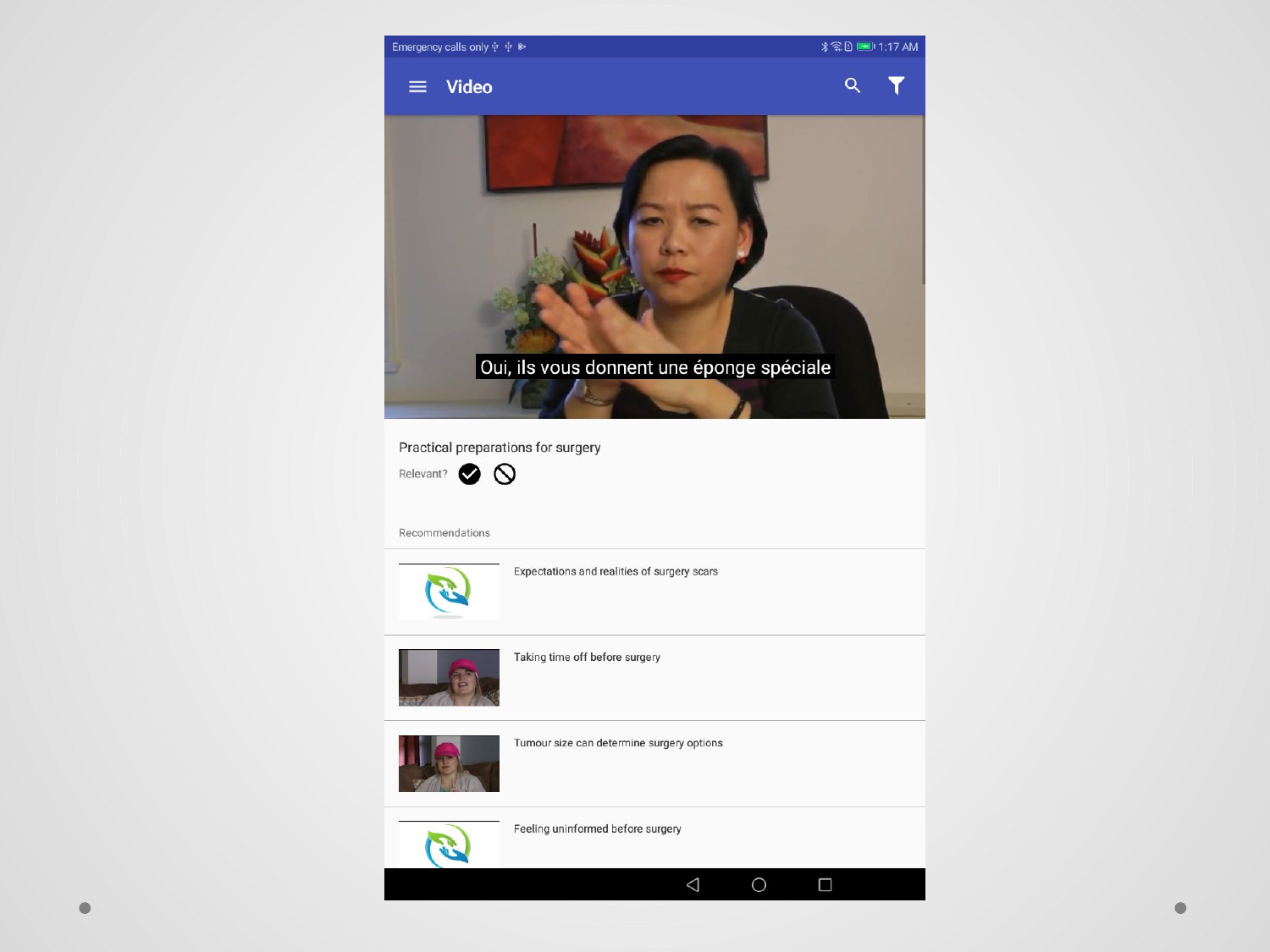

## Slide 15
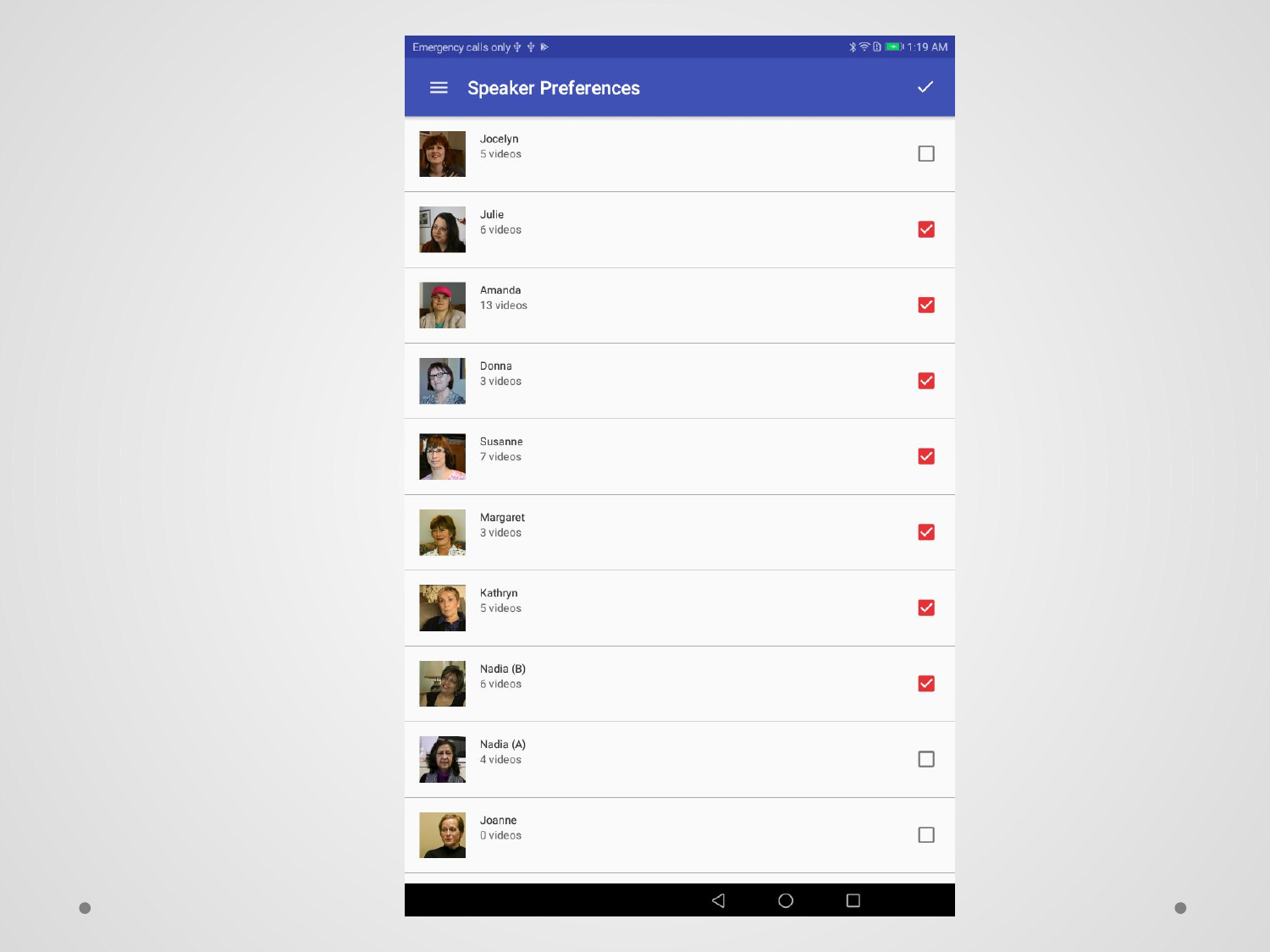

## Slide 16
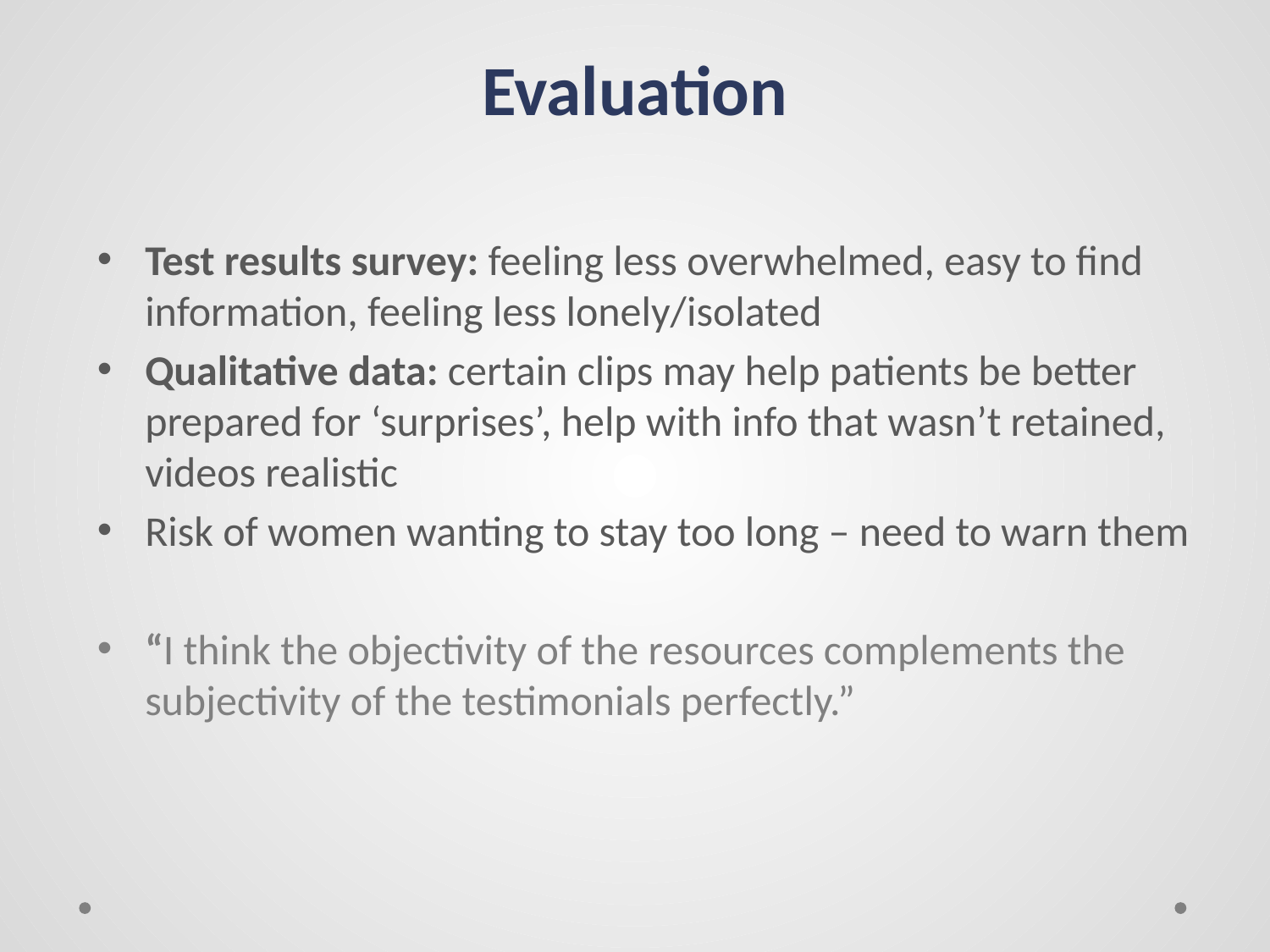

# Evaluation
Test results survey: feeling less overwhelmed, easy to find information, feeling less lonely/isolated
Qualitative data: certain clips may help patients be better prepared for ‘surprises’, help with info that wasn’t retained, videos realistic
Risk of women wanting to stay too long – need to warn them
“I think the objectivity of the resources complements the subjectivity of the testimonials perfectly.”

## Slide 17
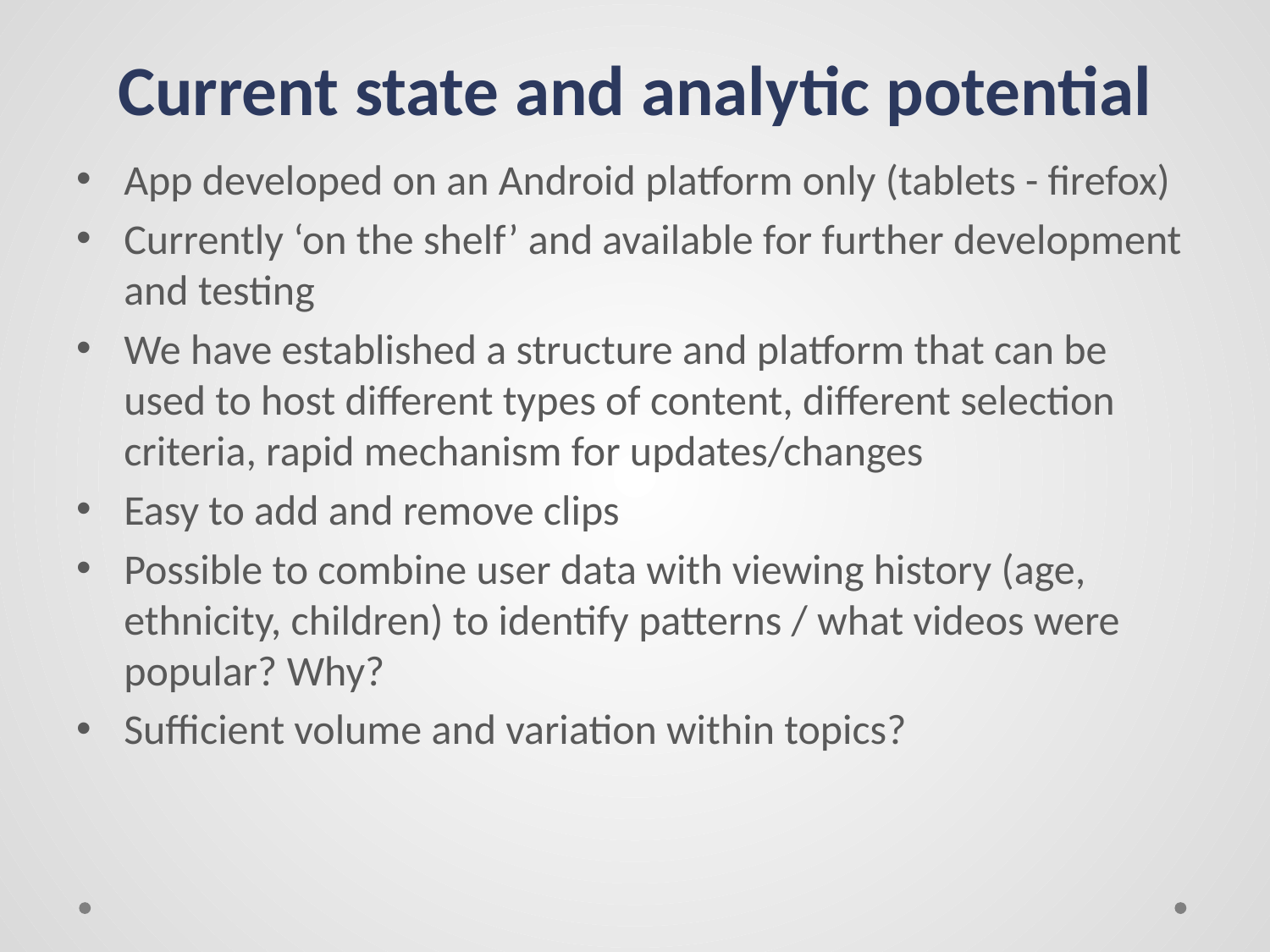

# Current state and analytic potential
App developed on an Android platform only (tablets - firefox)
Currently ‘on the shelf’ and available for further development and testing
We have established a structure and platform that can be used to host different types of content, different selection criteria, rapid mechanism for updates/changes
Easy to add and remove clips
Possible to combine user data with viewing history (age, ethnicity, children) to identify patterns / what videos were popular? Why?
Sufficient volume and variation within topics?

## Slide 18
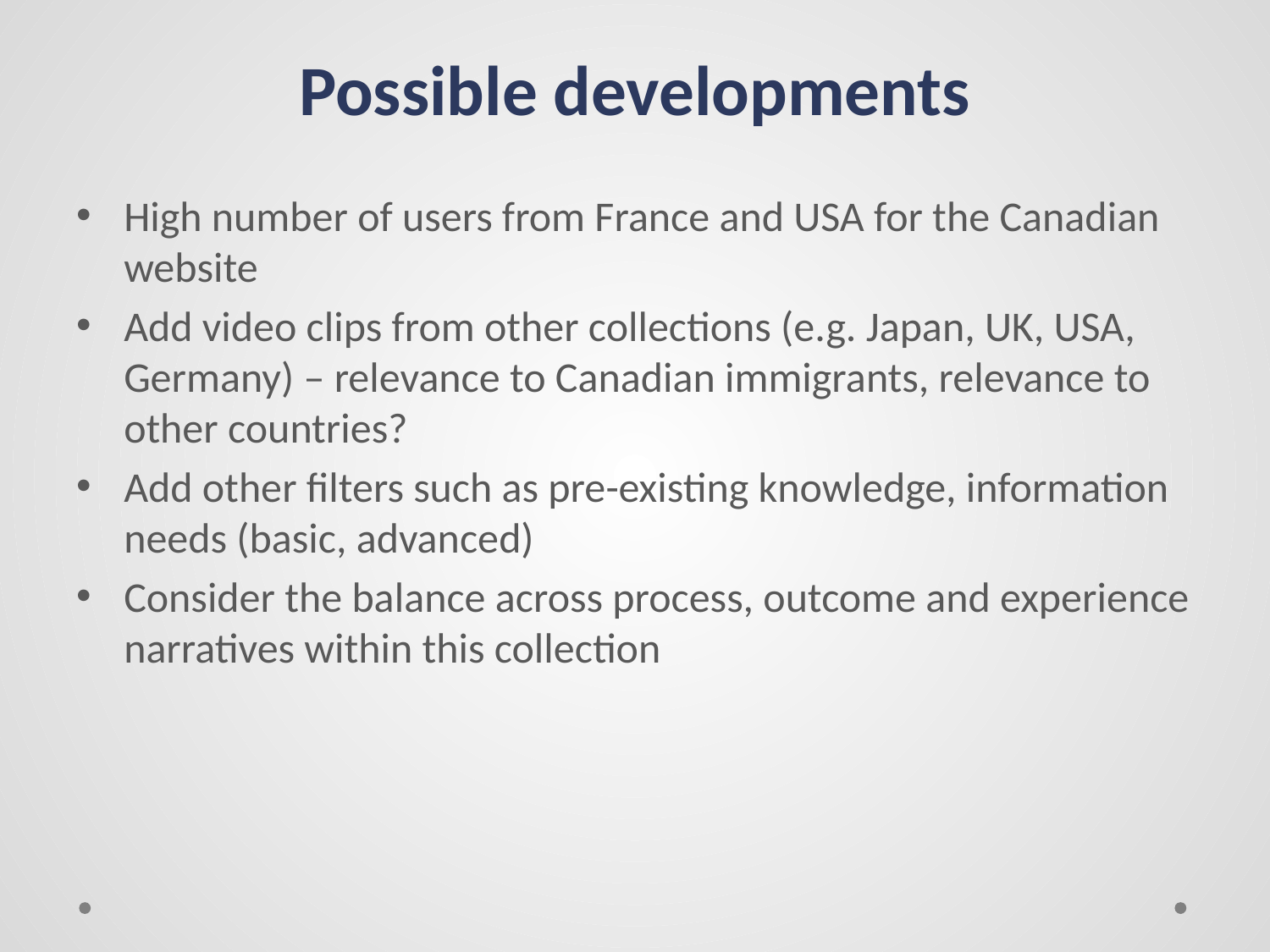

# Possible developments
High number of users from France and USA for the Canadian website
Add video clips from other collections (e.g. Japan, UK, USA, Germany) – relevance to Canadian immigrants, relevance to other countries?
Add other filters such as pre-existing knowledge, information needs (basic, advanced)
Consider the balance across process, outcome and experience narratives within this collection

## Slide 19
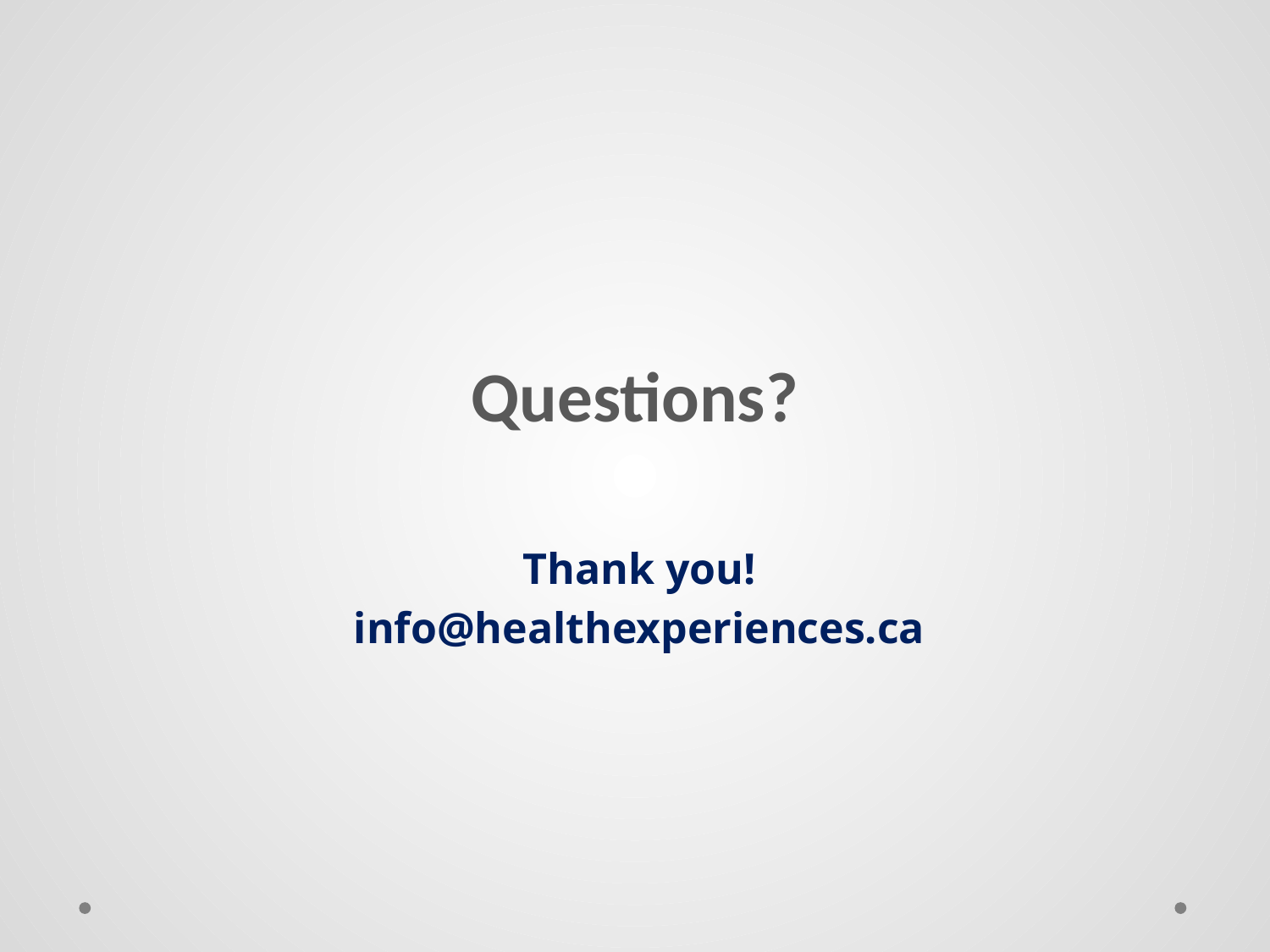

Questions?
Thank you!
info@healthexperiences.ca

## Slide 20
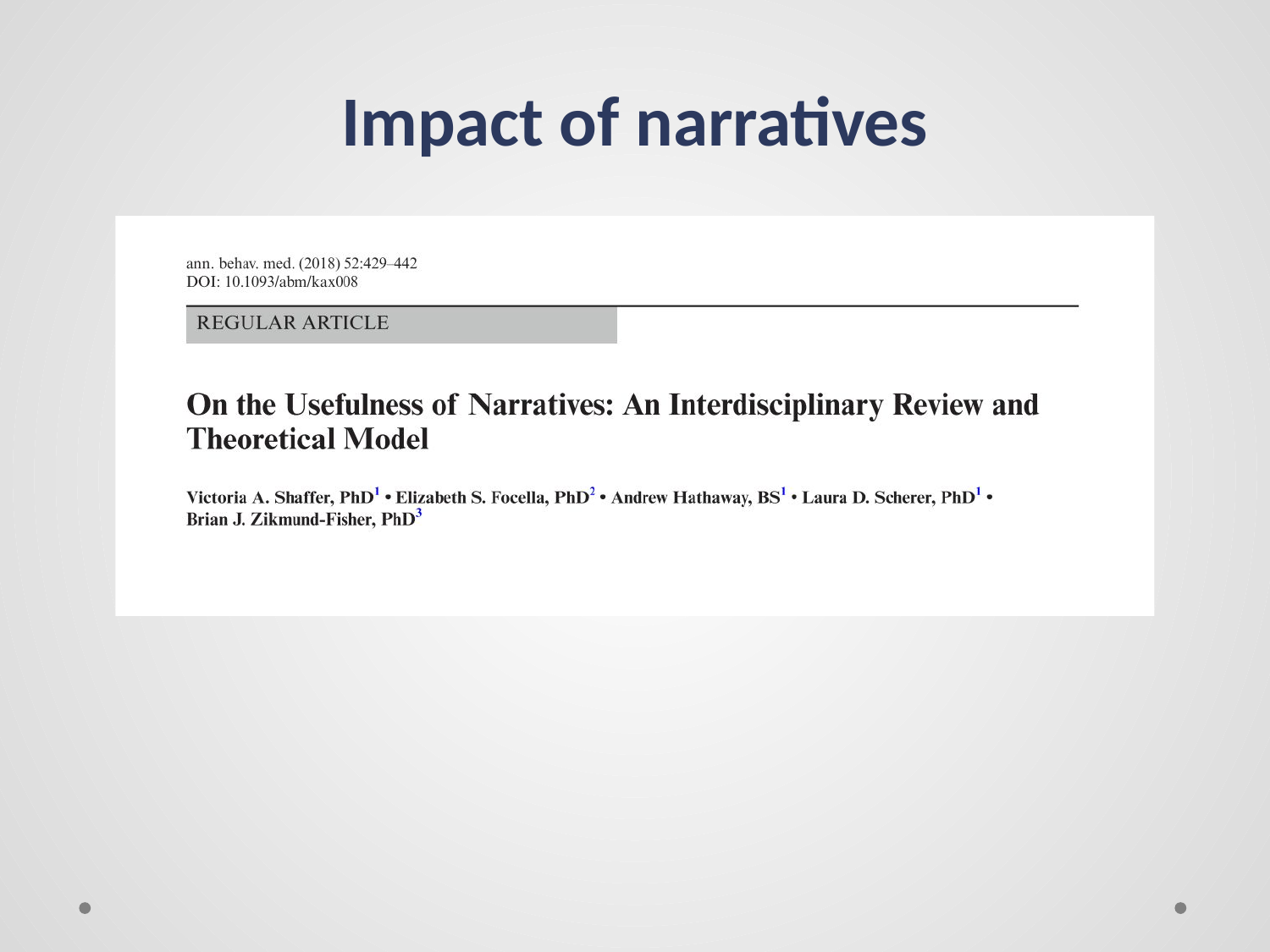

# Impact of narratives

## Slide 21
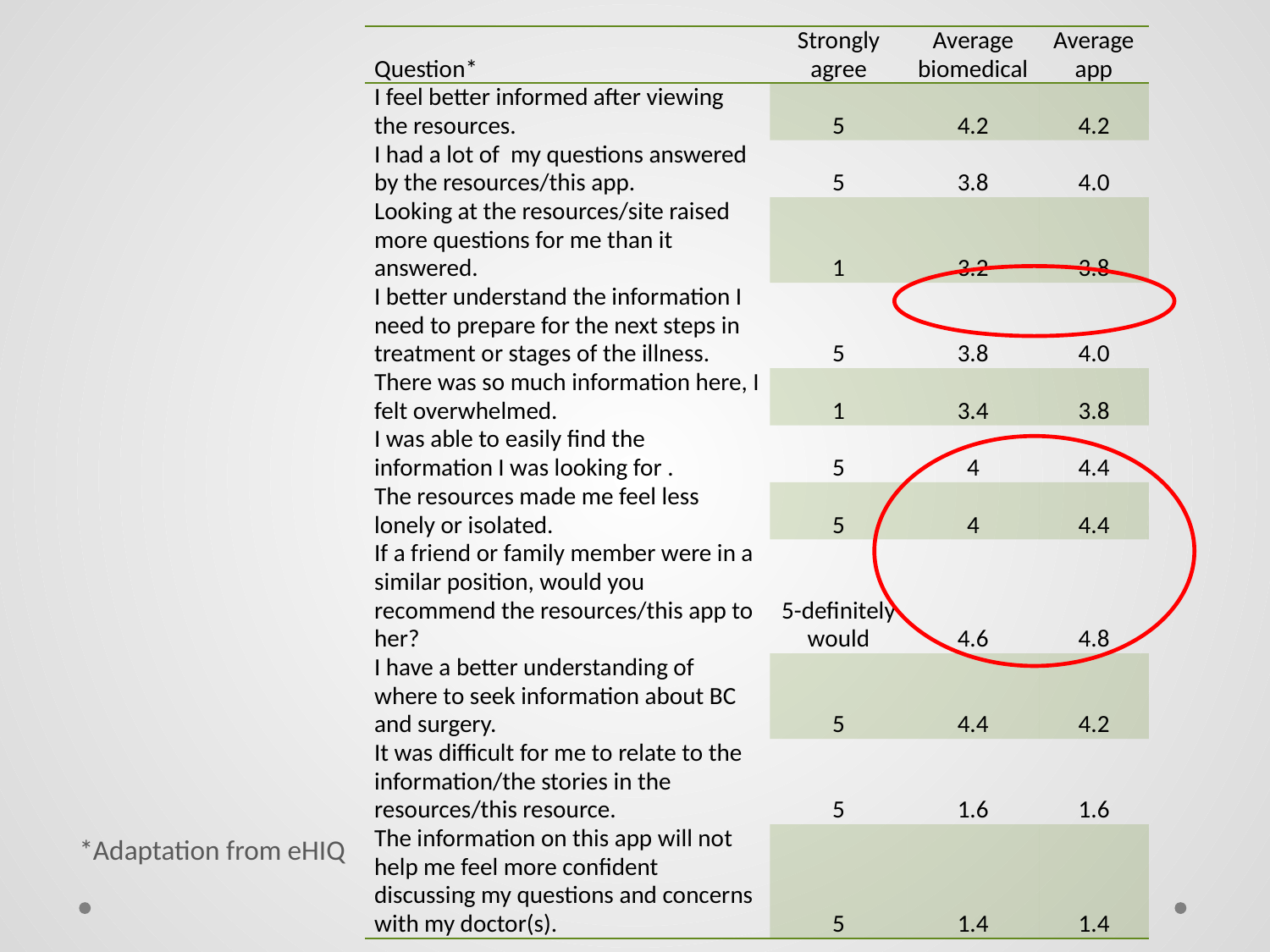

| Question\* | Strongly agree | Average biomedical | Average app |
| --- | --- | --- | --- |
| I feel better informed after viewing the resources. | 5 | 4.2 | 4.2 |
| I had a lot of my questions answered by the resources/this app. | 5 | 3.8 | 4.0 |
| Looking at the resources/site raised more questions for me than it answered. | 1 | 3.2 | 3.8 |
| I better understand the information I need to prepare for the next steps in treatment or stages of the illness. | 5 | 3.8 | 4.0 |
| There was so much information here, I felt overwhelmed. | 1 | 3.4 | 3.8 |
| I was able to easily find the information I was looking for . | 5 | 4 | 4.4 |
| The resources made me feel less lonely or isolated. | 5 | 4 | 4.4 |
| If a friend or family member were in a similar position, would you recommend the resources/this app to her? | 5-definitely would | 4.6 | 4.8 |
| I have a better understanding of where to seek information about BC and surgery. | 5 | 4.4 | 4.2 |
| It was difficult for me to relate to the information/the stories in the resources/this resource. | 5 | 1.6 | 1.6 |
| The information on this app will not help me feel more confident discussing my questions and concerns with my doctor(s). | 5 | 1.4 | 1.4 |
*Adaptation from eHIQ
